# Microglial Cytokines Mediate Plasticity Induced by 10 Hz Repetitive Magnetic Stimulation

**DOI:** 10.1523/JNEUROSCI.2226-22.2023

**Published:** 2023-04-26

**Authors:** Amelie Eichler, Dimitrios Kleidonas, Zsolt Turi, Maximilian Fliegauf, Matthias Kirsch, Dietmar Pfeifer, Takahiro Masuda, Marco Prinz, Maximilian Lenz, Andreas Vlachos

**Affiliations:** ^1^Department of Neuroanatomy, Institute of Anatomy and Cell Biology, Faculty of Medicine, University of Freiburg, 79104 Freiburg, Germany; ^2^Spemann Graduate School of Biology and Medicine, University of Freiburg, 79104 Freiburg, Germany; ^3^Faculty of Biology, University of Freiburg, 79104 Freiburg, Germany; ^4^Institute of Neuropathology, Faculty of Medicine, University of Freiburg, 79106 Freiburg, Germany; ^5^Department of Pharmaceutical Biology and Biotechnology, Institute of Pharmaceutical Sciences, University of Freiburg, 79104 Freiburg, Germany; ^6^Department of Hematology, Oncology and Stem Cell Transplantation, Medical Center, Faculty of Medicine, University of Freiburg, 79106 Freiburg, Germany; ^7^Department of Molecular and System Pharmacology, Graduate School of Pharmaceutical Sciences, Kyushu University, Fukuoka, 812-8582, Japan; ^8^Signalling Research Centres BIOSS and CIBSS, University of Freiburg, 79104 Freiburg, Germany; ^9^Center for Basics in Neuromodulation (NeuroModulBasics), Faculty of Medicine, University of Freiburg, 79106 Freiburg, Germany; ^10^Center BrainLinks-BrainTools, University of Freiburg, 79110 Freiburg, Germany

**Keywords:** excitatory synaptic plasticity, IL6, microglia, microglia depletion, rTMS, TNF

## Abstract

Microglia, the resident immune cells of the CNS, sense the activity of neurons and regulate physiological brain functions. They have been implicated in the pathology of brain diseases associated with alterations in neural excitability and plasticity. However, experimental and therapeutic approaches that modulate microglia function in a brain region-specific manner have not been established. In this study, we tested for the effects of repetitive transcranial magnetic stimulation (rTMS), a clinically used noninvasive brain stimulation technique, on microglia-mediated synaptic plasticity; 10 Hz electromagnetic stimulation triggered a release of plasticity-promoting cytokines from microglia in mouse organotypic brain tissue cultures of both sexes, while no significant changes in microglial morphology or microglia dynamics were observed. Indeed, substitution of tumor necrosis factor α (TNFα) and interleukin 6 (IL6) preserved synaptic plasticity induced by 10 Hz stimulation in the absence of microglia. Consistent with these findings, *in vivo* depletion of microglia abolished rTMS-induced changes in neurotransmission in the mPFC of anesthetized mice of both sexes. We conclude that rTMS affects neural excitability and plasticity by modulating the release of cytokines from microglia.

**SIGNIFICANCE STATEMENT** Repetitive transcranial magnetic stimulation (rTMS) is a noninvasive brain stimulation technique that induces cortical plasticity. Despite its wide use in neuroscience and clinical practice (e.g., depression treatment), the cellular and molecular mechanisms of rTMS-mediated plasticity remain not well understood. Herein, we report an important role of microglia and plasticity-promoting cytokines in synaptic plasticity induced by 10 Hz rTMS in organotypic slice cultures and anesthetized mice, thereby identifying microglia-mediated synaptic adaptation as a target of rTMS-based interventions.

## Introduction

Despite its increasingly prevalent clinical use, for example, for the treatment of patients with pharmaco-resistant depression, the cellular and molecular effects of repetitive transcranial magnetic stimulation (rTMS) remain poorly understood (Muller-Dahlhaus and Vlachos, 2013; Cirillo et al., 2017). It is widely known, however, that the electric fields produced by noninvasive magnetic brain stimulation modulate neural excitability and synaptic transmission in cortical circuits (Pell et al., 2011). These changes require neural activity (i.e., action potential [AP] induction) and Ca^2+^-dependent signaling pathways (i.e., NMDARs and L-type voltage-gated calcium channels) (Lenz et al., 2015). Such changes are consistent with the LTP of synaptic neurotransmission (Vlachos et al., 2012). It remains unclear, however, how rTMS and the induction of “LTP-like” plasticity assert positive effects in clinical settings.

Recent work suggests that microglia, the resident immune cells of the brain, sense and regulate synaptic transmission and plasticity (Schafer et al., 2012; Badimon et al., 2020; Nebeling et al., 2023). There is evidence that changes in network activity modulate microglia states (Pfeiffer et al., 2016; Stowell et al., 2019). These changes are reflected by the dynamic extension and retraction of microglial processes, by the formation of physical contacts between microglia and neurons, and by the activity-dependent secretion of plasticity-modulating factors (Akiyoshi et al., 2018; Sheppard et al., 2019; Cserep et al., 2020). Specifically, the plasticity-modulating effects of pro-inflammatory cytokines, such as TNFα and IL6, have been demonstrated in several experimental settings, both *in vitro* and *in vivo* (Stellwagen and Malenka, 2006; Santello et al., 2011; Heir and Stellwagen, 2020). These findings suggest that microglia play an important role in neural function and plasticity. Considering their role in pathologic brain states (Graeber and Streit, 2010; Prinz et al., 2019, 2021), it is interesting to theorize that the therapeutic effects of rTMS could, at least in part, depend on the modulation of microglia function. Indeed, evidence has been provided that rTMS affects microglial markers (Cullen and Young, 2016; Clarke et al., 2017; Stevanovic et al., 2019; Muri et al., 2020; Li et al., 2021). However, direct experimental evidence determining the role of microglia in rTMS-induced neural plasticity is currently not available.

To address the biological relevance of microglia in rTMS-induced synaptic plasticity, we depleted microglia from organotypic brain tissue cultures and the adult mouse brain and probed synaptic plasticity with a 10 Hz stimulation protocol. In turn, we tested for the effects of 10 Hz magnetic stimulation on the structural and functional properties of microglia and used *in vitro* cytokine release assays to identify the plasticity-promoting effects of microglial factors secreted after rTMS.

## Materials and Methods

### Ethics statement

Mice were maintained in a 12 h light/dark cycle with food and water available *ad libitum*. Every effort was made to minimize distress and pain of animals. All experimental procedures were performed according to German animal welfare legislation and approved by the competent authority (Regierungspräsidium Freiburg, G-20/154), appropriate animal welfare committee, and the animal welfare officer of the University of Freiburg, Faculty of Medicine (X-17/07K, X-17/09C, X-18/02C).

### Preparation of tissue cultures

Entorhino-hippocampal tissue cultures were prepared at postnatal days 3-5 from *C57BL/6J*, *HexB-tdTom* (Masuda et al., 2020) and *C57BL/6-Tg(TNFa-eGFP)* (Lenz et al., 2020) mice of either sex as previously described (Del Turco and Deller, 2007). Cultivation medium contained 50% (v/v) MEM, 25% (v/v) basal medium Eagle, 25% (v/v) heat-inactivated normal horse serum, 25 mm HEPES buffer solution, 0.15% (w/v) bicarbonate, 0.65% (w/v) glucose, 0.1 mg/ml streptomycin, 100 U/ml penicillin, and 2 mm GlutaMAX. The pH was adjusted to 7.3. All tissue cultures were allowed to mature for at least 18 d in a humidified atmosphere with 5% CO_2_ at 35°C. Cultivation medium was replaced 3 times per week.

### Microglia depletion *in vitro* and *in vivo*

Tissue cultures were treated immediately after preparation (DIV 0) with the colony-stimulating factor 1 receptor (CSF-1R) inhibitor PLX3397 (50 nm; #2501 Axon) for at least 18 d. Vehicle-only treated cultures (DMSO, 0.1 µl) served as age- and time-matched controls. For *in vivo* microglia depletion, we used the CSF-1R inhibitor BLZ945 (kindly provided by Novartis) dissolved in 20% (2-hydroxypropyl)-β-cyclodextrin (Sigma-Aldrich). A dose of 200 mg/kg body weight was applied by oral gavage in adult (8-week-old) mice for 7 consecutive days as previously described (Hagemeyer et al., 2017; Masuda et al., 2020). No weight loss or any apparent signs of stress could be detected throughout the treatment period. Vehicle-only treated and untreated animals were used as controls.

### rMS *in vitro*

To learn more about the cellular and molecular mechanisms of rTMS-induced plasticity, we established an *in vitro* model of r(T)MS using mouse entorhino-hippocampal slice cultures, which provide the advantage of a highly laminar fiberarchitecture and cytoarchitecture (Frotscher and Heimrich, 1995; Maus et al., 2020; Lenz et al., 2022); further advantages and disadvantages of these preparations were discussed previously (Vlachos et al., 2012). This approach allowed us to assess rMS-induced synaptic plasticity at the level of single identified neurons which are embedded in an organotypic neuronal network (compare Muller-Dahlhaus and Vlachos, 2013). Moreover, recent work confirmed the maintenance of microglia signatures and functions in organotypic slice cultures akin to what is seen *in vivo* (Delbridge et al., 2020). Tissue cultures (≥18 DIV) were transferred to a 35 mm Petri dish filled with prewarmed standard extracellular solution containing the following (in mm): 129 NaCl, 4 KCl, 1 MgCl_2_, 2 CaCl_2_, 4.2 glucose, 10 HEPES, 0.1 mg/ml streptomycin, 100 U/ml penicillin, pH 7.4 adjusted with NaOH, osmolarity adjusted with sucrose to 380-390 mOsm. Cultures were stimulated using the Magstim Rapid^2^ stimulator (Magstim) connected to a Double AirFilm Coil (coil parameters according to manufacturer's description: average inductance = 12 μH; pulse rise time ∼80 μs; pulse duration = 0.5 ms, biphasic; Magstim) with a biphasic current waveform. Cultures were positioned ∼1 cm under the center of the coil and oriented in a way that the induced electric field was parallel to the dendritic tree of CA1 pyramidal neurons. The stimulation protocol consisted of 900 pulses at 10 Hz (50% maximum stimulator output). Cultures were kept in the incubator for at least 2 h after stimulation before experimental assessment. Age- and time-matched control cultures were not stimulated but otherwise treated identically to stimulated cultures (sham stimulation). For cytokine substitution experiments, TNFα (5 ng/ml; #410-MT R&D Systems) and IL6 (2.5 ng/ml; #406-ML, R&D Systems) were added to the stimulation medium.

### rMS *in vivo*

rTMS was conducted in adult (∼8 weeks old) urethane-anesthetized (1.25 g kg^−1^, i.p.; 0.125 g kg^−1^, s.c.) *C57BL/6J* mice of either sex. The head was placed under the coil with the mPFC under the center. During the stimulation, the brain-to-coil distance was kept minimal, while brain-to-coil contact was avoided. Repetitive stimulation was performed at fixed intensities of 60% MSO (which corresponds to 90% motor threshold (see Lenz et al., 2016) using the same 10 Hz stimulation protocol described above. Control animals placed near the coil during stimulation were not stimulated but otherwise treated identically. All animals were transferred back to their cages with appropriate body temperature control and were held in anesthesia for 2 h under continuous surveillance. After the waiting period, ketamine/xylazine (100 mg kg^−1^/20 mg kg^−1^; i.p. application) was injected to achieve a suitable analgesia before rapid decapitation.

The brain was prepared as previously described (Ting et al., 2018; Lenz et al., 2021). After dissection, the brain was embedded in low-melting agar (1.8% w/v in PBS; Sigma-Aldrich #A9517) and frontal sections (350 µm thickness) containing the mPFC were prepared using a Leica VT1200S vibratome with a cutting angle of 15°. The brain was cut in NMDG-aCSF [containing the following (in mm): 92 NMDG, 2.5 KCl, 1.25 NaH_2_PO_4_, 30 NaHCO_3_, 20 HEPES, 25 glucose, 2 thiourea, 5 Na-ascorbate, 3 Na-pyruvate, 0.5 CaCl_2_, and 10 MgSO_4_, (pH 7.3-7.4)] at ∼0°C. After cutting, slices were recovered in NMDG-aCSF at 34°C. Sodium spike-in was performed according to a previously established protocol that is suitable for ∼8-week-old animals (Ting et al., 2018; Lenz et al., 2021). After recovery, we transferred the slices to a holding chamber [holding-aCSF; containing the following (in mm): 92 NaCl, 2.5 KCl, 1.25 NaH_2_PO_4_, 30 NaHCO_3_, 20 HEPES, 25 glucose, 2 thiourea, 5 Na-ascorbate, 3 Na-pyruvate, 2 CaCl_2_, and 2 MgSO_4,_ (pH 7.3-7.4)] at room temperature in which slices were maintained at least half an hour until electrophysiological assessment.

### Propidium iodide (PI) staining

Tissue cultures were incubated with PI (5 μg/ml; #P3566 Invitrogen) for 2 h, washed in PBS, and fixed as described below. Cultures treated for 4 h with NMDA (50 μg/ml; #0114 Tocris Bioscience) served as positive controls in these experiments. Cell nuclei were stained with DAPI, sections were mounted on microscope slides, and confocal images were acquired as described below.

### Immunostaining and imaging

Tissue cultures were fixed overnight in a solution of 4% (w/v) PFA in PBS (prepared from 16% PFA stocks in PBS according to the manufacturer's instruction; #28908 Fisher Scientific). After fixation, cultures were washed in PBS (0.1 m, pH 7.4) and consecutively incubated for 1 h with 10% (v/v) NGS in 0.5% (v/v) Triton X-100 containing PBS to reduce nonspecific staining and to increase antibody penetration. Subsequently, cultures were incubated overnight at 4°C with rabbit anti-Iba1 (1:1000; #019-19741 Fujifilm Wako) in PBS with 10% NGS and 0.1% Triton X-100. Sections were washed and incubated overnight at 4°C with appropriate AlexaFluor dye-conjugated secondary antibodies (1:1000, donkey anti-rabbit AlexaFluor-488 or -647; #A-21206 or #A-32795 Invitrogen) in PBS with 10% NGS or NHS, 0.1% Triton X-100. For *post hoc* visualization of patched pyramidal cells, Streptavidin AlexaFluor-488 or 633 (Streptavidin A488, 1:1000; #S32354 Invitrogen; Streptavidin A633, 1:1000; #S21375 Invitrogen) was added to the secondary antibody incubation solution. DAPI nuclear stain (1:2000 in PBS for 20 min; #62248 Fisher Scientific) was used to visualize cytoarchitecture. Cultures were washed, transferred onto glass slides, and mounted for visualization with DAKO anti-fading mounting medium (#S302380-2 Agilent).

**Figure 1.**
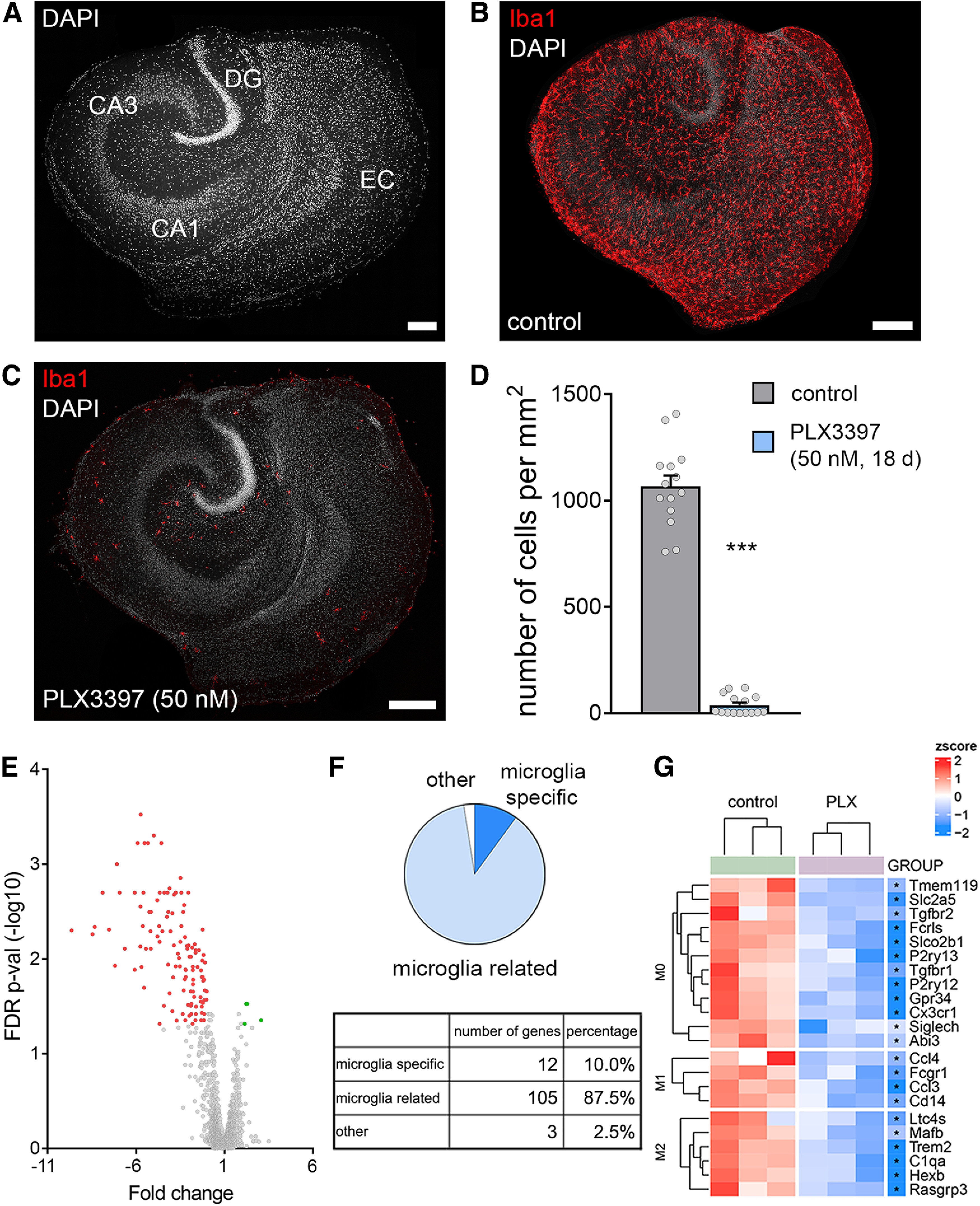
PLX3397 depletes microglia in organotypic tissue cultures. ***A***, Entorhino-hippocampal tissue culture stained with DAPI nuclear stain. EC, entorhinal cortex; DG, dentate gyrus. Scale bar, 200 µm. ***B***, ***C***, Representative examples of tissue cultures stained for the microglial marker Iba1. Note homogeneous distribution of microglia in the control culture and depletion of microglia following PLX3397 treatment (50 nm, 18 d). Scale bars, 200 µm. ***D***, Microglia cell counts in the respective groups (n_control_ = 14 cultures, n_PLX3397_ = 15 cultures; Mann–Whitney test, *U* = 0). ***E-G***, Affymetrix Microarray analysis of control cultures and cultures treated with PLX3397. ***E***, Volcano plot represents fold changes and FDR *p* values of analyzed transcripts. Green represents significantly upregulated transcripts. Red represents significantly downregulated transcripts. ***F***, Classification of differentially expressed transcripts; 97.5% of the differentially expressed transcripts are microglia-specific or microglia-related (for detailed results, see Extended Data Table 1-1). ***G***, Hierarchical clustering of differentially expressed gene sets characteristic of M0-, M1-, and M2-classified microglia. Each sample consisted of three pooled cultures (*n* = 3 samples in each group). Colored dots represent individual data points. Data are mean ± SEM. ***p < 0.001.

A Leica SP8 laser scanning microscope equipped with a 20× multi-immersion (NA 0.75; Leica), a 40× oil-immersion (NA 1.30; Leica) and a 63× oil-immersion objective (NA 1.40; Leica) was used for confocal image acquisition. Images for analysis of microglia cell density (see Fig. 1) and images of PI stainings (see Fig. 4) were acquired with a 20× objective at 0.75× optical zoom (resolution: 512 × 512 pixels). Image stacks for spine density and spine volume analysis (see Fig. 6) were acquired with a 63× oil-immersion objective at 5.0× optical zoom (resolution: 1024 × 1024, Δz = 0.22 µm at ideal Nyquist rate). Image stacks of Iba1-stained *HexB-tdTom* cultures (see Fig. 8) were acquired with a 20× objective at 2.0× optical zoom (resolution: 512 × 512 pixels). Image stacks of Iba1-stained acute cortical slices (see Fig. 13) were acquired using a 40× oil-immersion objective at 2.0× optical zoom (resolution 1024 × 1024, Δz = 1 µm). Laser intensity and detector gain were set to achieve comparable overall fluorescence intensity throughout stacks between all groups in each experimental setting.

### Live-cell imaging

Live-cell imaging of tissue cultures was performed at a Zeiss LSM800 microscope equipped with a 10× water immersion (NA 0.3; Carl Zeiss) and a 40× water immersion objective (NA 1.0; Carl Zeiss). Filter membranes with 2-6 cultures were placed in a 35 mm Petri Dish containing pre-oxygenated imaging solution consisting of 50% (v/v) MEM, 25% (v/v) basal medium Eagle, 50 mm HEPES buffer solution (25% v/v), 0.65% (w/v) glucose, 0.15% (w/v) bicarbonate, 0.1 mg/ml streptomycin, 100 U/ml penicillin, 2 mm GlutaMAX, and 0.1 mm trolox. The cultures were kept at 35°C during the imaging procedure.

Live-cell imaging of homozygous (*HexB^tdT/tdT^*) and heterozygous (*HexB*^*tdT*/+^) cultures prepared from *HexB-tdTom* transgenic animals was performed to assess microglia morphology after rMS (see Fig. 8). Cultures were stimulated as described above (rMS and sham stimulation), and imaging was started immediately in imaging solution under continuous oxygenation (5% CO_2_/95% O_2_). For 3 h, every 2 min a *z* stack of the same cell was recorded using a 40× water immersion objective with Δz = 1 μm at ideal Nyquist rate and an optical zoom of 1.0× (resolution 512 × 512 pixels, 2× line average). Laser intensity and detector gain were initially set and were kept constant over image acquisition time.

Live-cell imaging of *C57BL/6-Tg(TNFa-eGFP)* cultures was performed to monitor TNFα expression after rMS as an indicator of neuroinflammation (see Fig. 9). Cultures were stimulated as described above (rMS and sham stimulation) and kept in the incubator after stimulation. After 3 h, a *z* stack of each culture was recorded using a 10× water immersion objective with Δz = 6.3 μm at ideal Nyquist rate and an optical zoom of 0.5× (resolution 1024 × 1024 pixels). Laser intensity and detector gain were initially set to keep the fluorescent signal in a dynamic range throughout the experiment and were kept constant. Confocal image stacks were stored as .czi files.

### Transcriptome microarray

Tissue cultures that were cultivated on one filter membrane (three cultures) were transferred as one sample into RLT buffer (QIAGEN), and RNA was isolated according to the manufacturer's instructions (RNeasy Plus Micro Kit; #74034 QIAGEN). RNA was eluted in 50 µl water and precipitated in 0.75 m ammonium-acetate and 10 µg glycogen (#R0551, Fisher Scientific) by adding 125 µl ethanol (100%). Samples were incubated at −80°C overnight and consecutively centrifuged for 30 min at 4°C. Pellets were washed with 70% ethanol, centrifuged again and dried. Finally, pellets were dissolved in water for further processing. RNA concentration and integrity were consecutively analyzed by capillary electrophoresis using a Fragment Analyser (Advanced Analytical Technologies). RNA samples with RNA quality numbers (RQN) > 8.0 were further processed with the Affymetrix WT Plus kit and hybridized to Clariom S mouse arrays as described by the manufacturer (Fisher Scientific). Briefly, labeled fragments were hybridized to arrays for 16 h at 45°C, 60 rpm in a GeneChip Hybridization Oven (Fisher Scientific). After washing and staining, the arrays were scanned with the Affymetrix GeneChip Scanner 3000 7G (Fisher Scientific). CEL files were produced from the raw data with Affymetrix GeneChip Command Console Software Version 4.1.2 (Fisher Scientific).

### Cytokine detection assay

To analyze protein release on rMS, cultures were stimulated on incubation medium in interface configuration with three cultures grown on one filter membrane. To test for activity-induced cytokine release during stimulation, we added the voltage-gated sodium channel inhibitor tetrodotoxin (2 μm; #18660-81-6 Biotrend) to the incubation medium during stimulation in some experiments. Three hours after stimulation, both incubation medium (for detection of protein release) and tissue cultures (for gene expression analysis) were collected and frozen in liquid nitrogen until further processing.

For cytokine detection, a V-Plex Proinflammatory Panel 1 (mouse) Kit Plus (#K15048G Mesoscale Discovery) was used. The collected incubation medium was diluted 1:1 in diluent provided with the kit. Protein detection was performed according to the manufacturer's instructions. A precoated plate with capture antibodies on defined spots was incubated with the diluted samples overnight. After washing, samples were incubated overnight with a solution containing electrochemiluminescent MSD SULFO-TAG detection antibodies (Mesoscale Discovery; Antibodies: Anti-ms TNFα Antibody #D22QW, Anti-ms IL6 Antibody #D22QX, Anti-ms CXCL1 Antibody #D22QT). After washing, samples were measured with a MESO QuickPlex SQ 120 instrument (Mesoscale Discovery). The respective protein concentrations were determined using the MSD Discovery Workbench software (Mesoscale Discovery).

### RNA isolation and qRT-PCR

RNA isolation for qPCR analysis was performed as follows: Tissue cultures that were cultivated on one filter membrane (three cultures) were transferred as one sample into RNA Protection buffer (New England Biolabs), and RNA was isolated according to the manufacturer's instructions (Monarch Total RNA Miniprep Kit; #T2010S New England Biolabs). As a quality control, the RNA integrity number (RIN) value of RNA isolated from tissue culture was determined using the Agilent RNA 6000 Pico Kit (#5067-1513 Agilent) with a 2100 Bioanalyzer (#G2939BA Agilent; Mean RIN value: 8.9). Purified RNA was consecutively reverse transcribed (RevertAid RT Kit; **#**K1691 Fisher Scientific). cDNA was diluted in water to a final concentration of 3 ng/ml. qRT-PCR was performed using a C1000 Touch Thermal Cycler (Bio-Rad) and the CFX 384 Real-Time PCR system (Bio-Rad). 13.5 ng target cDNA diluted in TaqMan Gene Expression Master Mix (#4369016 Applied Biosystems) was amplified using standard TaqMan gene expression assays (Applied Biosystems; Assay-IDs: *Gapdh*: Mm99999915_g1; *Tnf*: Mm00443258_m1; *Il6*: Mm00446190_m1; *Cxcl1*: Mm04207460_m1). The qRT-PCR protocol was performed as follows: 1 cycle of 50°C for 2 min, 1 cycle of 95°C for 10 min, 40 cycles of 95°C for 15 s, and 60°C for 1 min. Three technical replicates of each sample were used and no amplification was detected in nontemplate controls. Amplification curves were excluded from further analysis if efficiency values were <90 or exceeded 110 according to automated calculation by the Bio-Rad CFX Maestro software package. Data were exported and stored on a computer as .pcrd-files.

### Whole-cell patch-clamp recordings

Whole-cell patch-clamp recordings were conducted at 35°C (3-6 neurons per culture). Patch pipettes contained the following (in mm): 126 K-gluconate, 10 HEPES, 4 KCl, 4 ATP-Mg, 0.3 GTP-Na_2_, 10 PO-creatine, 0.3% (w/v) biocytin (pH 7.25 with KOH, 290 mOsm with sucrose), having a tip resistance of 4-6 mΩ. Pyramidal neurons were visually identified using an LN-Scope (Luigs & Neumann) equipped with an infrared dot-contrast and a 40× water immersion objective (NA 0.8; Olympus). Electrophysiological signals were amplified using a Multiclamp 700B amplifier, digitized with a Digidata 1550B digitizer, and visualized with the pClamp 11 software package.

For whole-cell patch-clamp recordings of CA1 pyramidal neurons in tissue cultures, the bath solution contained the following (in mm): 126 NaCl, 2.5 KCl, 26 NaHCO_3_, 1.25 NaH_2_PO_4_, 2 CaCl_2_, 2 MgCl_2_, and 10 glucose (aCSF) and was oxygenated continuously (5% CO_2_/95% O_2_). Spontaneous EPSCs (sEPSCs) of CA1 pyramidal neurons were recorded in voltage-clamp mode at a holding potential of −60 mV. Series resistance was monitored before and after each recording, and recordings were discarded if the series resistance reached ≥30 mΩ. For miniature EPSC (mEPSC) recordings of CA1 pyramidal neurons, D-APV (10 μm; #ab120003 Abcam), (–)-bicuculline-methiodide (10 μm; #ab120108 Abcam), and TTX (0.5 μm; #18 660-81-6 Biotrend) were added to the external solution.

Whole-cell patch-clamp recordings of superficial (layer 2/3) cortical pyramidal neurons in acute mouse brain slices were conducted in a bath solution containing holding-aCSF. For sEPSC recordings, layer 2/3 pyramidal neurons were held at −70 mV in voltage-clamp mode.

For recording of intrinsic cellular properties in current-clamp mode, pipette capacitance of 2.0 pF was corrected and series resistance was compensated using the automated bridge balance tool of the MultiClamp commander. *I–V* curves were generated by injecting 1 s square pulse currents starting at −100 pA and increasing in 10 pA steps (sweep duration: 2 s). Series resistance was monitored, and recordings were discarded if the series resistance reached ≥30 mΩ.

### Multiscale modeling

A three-dimensional mesh model was created with two compartments (i.e., bath solution and organotypic tissue culture) using the finite element method and the program Gmsh (4.8.4). Local mesh resolution was increased from 0.01 to 0.004 units in the CA1 region of the culture (i.e., ROI) where neurons were placed. The final mesh consisted of 3.55 × 10^6^ nodes and 2.11 × 10^7^ tetrahedrons. The mean tetrahedron edge length was 5.6 µm in the ROI. The physical dimensions of the mesh model were adapted from the *in vitro* setting.

The coil-to-culture distance was kept at 10 mm, and the coil was positioned above the culture. Electrical conductivities for the bath and culture were 1.654 and 0.275 S m^−1^, respectively. The rate of change of the coil current was set to 1.4 A µs^−1^ at 1% MSO, and it was scaled up to 50% MSO. Macroscopic electric field simulations were performed using SimNIBS (3.2.4) and MATLAB (2020b). A validated 70 mm MagStim figure-of-eight coil was used in all simulations.

For multiscale modeling, we used the Neuron Modeling for TMS (NeMo-TMS) framework to study the biological responses of CA1 pyramidal neurons to biphasic single-pulse TMS and rTMS (Shirinpour et al., 2021). Axonal morphology was adopted from an example cell (Shirinpour et al., 2021). For all neurons, we implemented the generalized version of the Jarsky model (Shirinpour et al., 2021).

We extracted the membrane potentials and voltage-gated calcium “influx” from the somatic and dendritic compartments (Shirinpour et al., 2021). We analyzed the number of APs, calcium spikes, and their peak values. Simulations were run on a high-performance computer in the state of Baden-Württemberg, Germany (bwHPC).

### Experimental design and statistical analysis

#### Study design

In this study, we used age-matched organotypic entorhino-hippocampal tissue cultures and ∼8-week-old adult mice of either sex in a prospective study design. Treatment with PLX3397 was used to deplete microglia from organotypic tissue cultures. Vehicle-only treated, age-matched cultures served as controls in these experiments. For microglia depletion in adult mice (∼8 weeks old), treatment with BLZ945 was used. Nontreated and vehicle-only treated mice of the same age were used as controls in these experiments. Excitatory inputs onto pyramidal neurons were evaluated in age-matched tissue cultures or ∼8-week-old adult mice after rMS. Sham-stimulated age-matched tissue cultures or ∼8-week-old mice served as controls. In experiments that included treatment with TTX, TNFα, or IL6, vehicle-only treatment served as control.

#### Quantification

For the analysis of microglia cell density and spine density, cells or spines were counted manually in maximum intensity projections of the confocal image stacks using the “Cell Counter” plugin of Fiji image processing package (available at https://fiji.sc/) (Schindelin et al., 2012).

Spine head volumes were assessed in the confocal image stacks using the Imaris x64 (version 9.5.0) software. The surface tool with the “split touching object” option enabled was used to measure the volume of spine heads. Files were stored as .ims.

Confocal images of PI-stained cultures were processed and analyzed using the Fiji image processing package. After background subtraction (rolling ball radius 50 pixels), images were binarized and PI-positive particles were displayed and counted using the “Analyze Particles” function. Values were normalized to the mean value of the control group.

Confocal image stacks of heterozygous *C57BL/6-Tg(TNFa-eGFP)* cultures were processed and analyzed as previously described (Lenz et al., 2020) using the Fiji image processing package. Mean fluorescence intensity of the culture area was normalized to the mean value of fluorescence intensity of the sham-stimulated cultures.

Dendritic morphologies were assessed using the Neurolucida 360 software (version 2020.1.1). Cells were semiautomatically reconstructed with the “user guided tree reconstruction” function. Reconstructions were saved as .DAT files, and analysis was performed in the Neurolucida Explorer (version 2019.2.1).

To analyze microglia morphology in *HexB-tdTom* cultures, confocal image stacks were processed and analyzed using the Fiji image processing package. Of each *z* stack, a maximum intensity projection was generated and binarized using the “Trainable Weka Segmentation” plugin (Arganda-Carreras et al., 2017). The same classifier was applied to all images of the same microglia over the recorded 3 h. After removing outliers (radius = 2 pixels, threshold = 50), microglia scanning density and microglia domain of each image were manually assessed as previously described (Pfeiffer et al., 2016). Values were normalized to the mean.

For the analysis of microglial morphology in acute mouse cortical slices, stacks of single cells were also processed and analyzed using the Fiji image processing package. First, each stack was processed using the “despeckle” function, then a maximum intensity projection was generated. After removing outliers (radius = 3 pixels, threshold = 50), the image was binarized as described before. Again, outliers were removed (radius = 2 pixels, threshold = 50). Microglia scanning density and microglia domain of each image were manually assessed.

Single-cell recordings were analyzed offline using Clampfit 11 of the pClamp11 software package (Molecular Devices). sEPSC and mEPSC properties were analyzed using the automated template search tool for event detection (Lenz et al., 2021). Input resistance was calculated for the injection of −100 pA current at a time frame of 200 ms with maximum distance to the Sag current. Resting membrane potential was calculated as the mean baseline value. AP detection was performed using the input/output curve threshold search event detection tool, and the AP frequency was assessed on the number of APs detected during the respective injection step. Four cells in the microglia-depleted (BLZ945-treated) sham-stimulated group were excluded in the analysis of intrinsic properties (see Fig. 13) because of loss of the integrity of the patch during recording of the *I–V* curve. In the control group data of vehicle-only treated and untreated animals were pooled (see Fig. 13).

Affymetrix GeneChip microarray data (CEL files) were analyzed using the Affymetrix Transcriptome Analysis Console (TAC version 4.0.2.15). Gene expression was considered significantly different when FDR *p* < 0.05 and fold change <−2 or >2. Differentially expressed well-annotated genes were considered “microglia-specific” (see Fig. 1) if they were part of highly specific microglia markers found by Chiu et al. (2013) and “microglia-related” if expression of these genes in microglia was reported in the literature elsewhere. A full list of differentially expressed genes, including predicted genes, is provided in Extended Data Table 1-1.

10.1523/JNEUROSCI.2226-22.2023.tab1-1 Table 1-1 : Microarray analysis of differentially expressed microglia-related or -specific genes. Download Table 1-1, DOCX file.

qRT-PCR data were analyzed as previously described (Lenz et al., 2020) with the Bio-Rad CFX Maestro 1.0 software package using the ΔΔCq method with *Gapdh* as reference gene. Values were normalized to the mean value of the respective vehicle-treated control group.

Mesoscale cytokine detection assay was analyzed using the MSD Discovery Workbench 4.0 platform. mRNA/protein level correlations were visualized by a linear regression fit and analyzed using nonparametric Spearman's correlation coefficients (*r*).

#### Statistical analysis

Statistical evaluation of the multiscale modeling was implemented in R (4.0.3; https://www.R-project.org/) and R Studio (1.3.1093; http://www.rstudio.com/) integrated development environment. We ran generalized linear mixed models (GLMMs) with predictors Treatment (two levels: control, PLX3397) and Compartment (three levels: soma, apical, and basal dendrites). GLMMs allow modeling-dependent variables from different distributions and model both fixed and random effects (Stroup, 2012). The null model contained the cell as random intercept, and we added each predictor and their interaction terms one-by-one to the subsequent models. The Bayesian information criterion (BIC) was used to compare the current model with the previous one. We selected the winning model if the ΔBIC was at least 10 units for the null or previous model (Kass and Raftery, 1995; Anderson and Burnham, 2004; Fabozzi, 2014).

Data were analyzed using GraphPad Prism 7 and 9 (GraphPad Software). Statistical comparisons were made using nonparametric tests, since normal distribution of data could not be assured. For column statistics, Mann–Whitney test (to compare two groups) and Kruskal–Wallis test followed by Dunn's multiple comparisons (to compare three groups) were used. For statistical comparison of *XY*-plots, we used a repeated-measures two-way ANOVA test (repeated measurements/analysis) with Sidak's multiple comparisons. *p* < 0.05 was considered a significant difference. In the text and figures, values represent mean ± SEM. *N* numbers are provided in the figure legends. Statistical differences in *XY* plots are indicated in the legend or the title of the figure panels when detected through multiple comparisons.

### Data and materials availability

Source data with statistical evaluations are provided on the Dryad data repository (doi: 10.5061/dryad.ngf1vhhzk). Modeling data and neuronal morphologies are available on GitHub (https://github.com/ZsoltTuri/2023_microglia_10Hz_rMS). Original data are available from the corresponding authors on reasonable request.

### Digital illustrations

Figures were prepared using Photoshop graphics software (Adobe). Image brightness and contrast were adjusted.

## Results

### Pharmacologic depletion of microglia in organotypic tissue cultures

To determine the role of microglia in r(T)MS-induced synaptic plasticity, organotypic tissue cultures were treated with the CSF-1R antagonist PLX3397, which readily depletes microglia (Elmore et al., 2014; Coleman et al., 2020). Tissue cultures containing the entorhinal cortex and the hippocampus were exposed to 50 nm PLX3397 for at least 18 DIV, starting immediately after tissue culture preparation (Fig. 1). A robust and almost complete depletion of microglia (∼96% reduction in cell density) was observed in the 3-week-old tissue cultures, as demonstrated by immunostaining for the microglial marker Iba1 (Fig. 1*B–D*, Mann–Whitney test, *p* < 0.001, *U* = 0).

Successful depletion of microglia was also confirmed with RNA microarray analysis (Fig. 1*E–G*). We observed a significant reduction of microglia-specific and microglia-related genes in the group treated with PLX3397 (Fig. 1*E*,*F*). Specifically, gene sets characteristic of M0-, M1-, and M2-classified microglia (Jurga et al., 2020) were reduced in our tissue cultures (Fig. 1*G*). In turn, no major changes in the expression of genes related to astrocytes, oligodendrocytes, and neurons (i.e., synaptic genes were observed following microglia depletion). These findings show that treatment with PLX3397 results in a robust and specific depletion of microglia in 3-week-old organotypic tissue cultures.

### CA1 pyramidal neurons in microglia-depleted tissue cultures do not express rMS-induced synaptic plasticity

**Figure 2.**
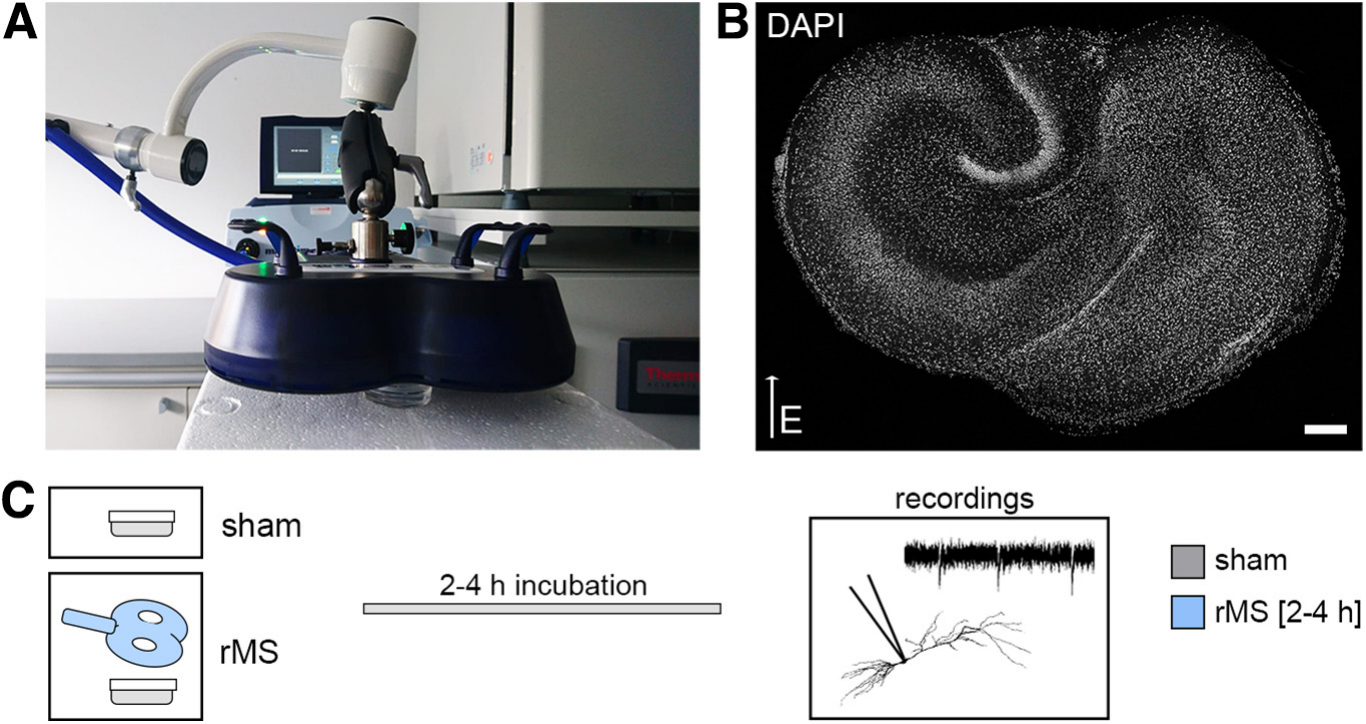
10 Hz rMS in organotypic tissue cultures. ***A***, ***B***, Entorhino-hippocampal tissue cultures (DAPI nuclear stain, ***B***) were stimulated with a 70 mm outer wing diameter figure-of-eight coil (Magstim). Filter inserts carrying 2-6 tissue cultures were placed in a Petri dish below the coil. The orientation within the electromagnetic field and distance to the coil was kept constant in all experiments (DAPI nuclear stain). Scale bar, 200 µm. ***C***, Sham-stimulated cultures were not stimulated but otherwise treated equally. After stimulation, cultures were kept in the incubator for 2-4 h before further experimental procedures, such as patch-clamp recordings.

**Figure 3.**
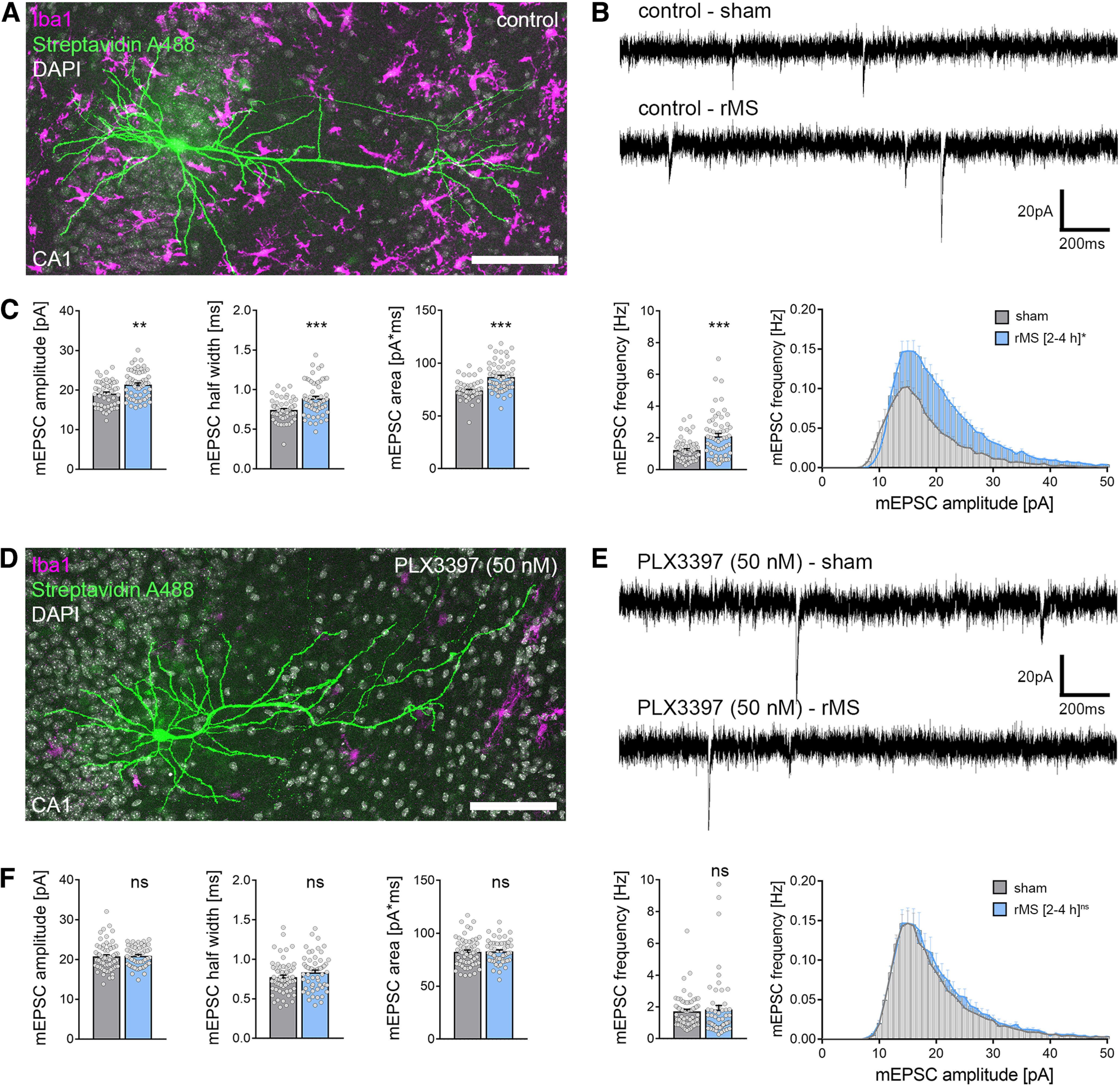
CA1 pyramidal neurons in microglia-depleted tissue cultures do not express excitatory synaptic plasticity induced by 10 Hz rMS. ***A***, Example of a recorded and *post hoc* stained CA1 pyramidal neuron in a tissue culture stained with the microglial marker Iba1. Scale bar, 100 µm. ***B***, ***C***, Sample traces and group data of AMPAR-mediated mEPSCs recorded from CA1 pyramidal neurons in sham-stimulated and 10 Hz rMS-stimulated cultures 2-4 h after stimulation (n_control-sham_ = 56 cells, n_control-rMS_ = 60 cells; Mann–Whitney test, U_amplitude_ = 1091, U_half-width_ = 1042, U_area_ = 956, U_frequency_ = 651; repeated-measures two-way ANOVA followed by Sidak's multiple comparisons test for amplitude-frequency-plot). ***D***, Example of a recorded and *post hoc* stained CA1 pyramidal neuron in a PLX3397-treated, microglia-depleted tissue culture stained with the microglial marker Iba1. Scale bar, 100 µm. ***E***, ***F***, Representative traces and group data of AMPAR-mediated mEPSCs recorded from CA1 pyramidal cells in sham-stimulated and 10 Hz rMS-stimulated microglia-depleted tissue cultures 2-4 h after stimulation (n_PLX3397-sham_ = 61 cells, n_PLX3397-rMS_ = 55 cells; Mann–Whitney test; repeated measures two-way ANOVA followed by Sidak's multiple comparisons test for amplitude-frequency plot). Gray dots represent individual data points. Data are mean ± SEM. **p < 0.01. ***p < 0.001.

Microglia-depleted tissue cultures and nondepleted control cultures were stimulated with a 10 Hz stimulation protocol consisting of 900 pulses at 50% maximum stimulator output using a Magstim Rapid^2^ stimulator equipped with an AirFilm coil (Fig. 2). The distance from the coil and the orientation of the stimulated tissue within the electric field were kept constant in all experiments (Fig. 2*A*,*B*). CA1 pyramidal neurons were patched, and AMPAR-mediated mEPSCs were recorded 2-4 h after stimulation (Fig. 2*C*). Sham-stimulated controls were treated the same way, except for stimulation. In line with previous work (Vlachos et al., 2012; Lenz et al., 2015, 2020), 10 Hz rMS induced a robust strengthening of excitatory synapses in CA1 pyramidal neurons of control tissue cultures, as demonstrated by increased mean mEPSC amplitude, half-width, area, and frequency (Fig. 3*A–C*; Mann–Whitney test; mEPSC amplitude: *p* = 0.001, *U* = 1091; mEPSC half-width: *p* < 0.001, *U* = 956; mEPSC area: *p* < 0.001, *U* = 651; mEPSC frequency: *p* < 0.001, *U* = 1042; amplitude-frequency plot: repeated-measures two-way ANOVA with Sidak's multiple comparisons, *p*_stimulation_<0.001, *F* = 16.96; *p*_mEPSC amplitude bin_< 0.001, *F* = 113.7). Conversely, no changes in mEPSCs were observed in microglia-depleted tissue cultures following rMS (Fig. 3*D*,*E*; Mann–Whitney test; mEPSC amplitude: *p* = 0.46, *U* = 1542; mEPSC half-width: *p* = 0.13, *U* = 1403; mEPSC area: *p* = 0.72, *U* = 1612; mEPSC frequency: *p* = 0.08, *U* = 1362; amplitude-frequency plot: repeated measures two-way ANOVA with Sidak's multiple comparisons, *p*_stimulation_ = 0.59, *F* = 0.30; *p*_mEPSC amplitude bin_< 0.001, *F* = 107.1). These results demonstrate that the presence of microglia is required for rMS-induced synaptic plasticity.

### Microglia depletion does not affect cell viability

**Figure 4.**
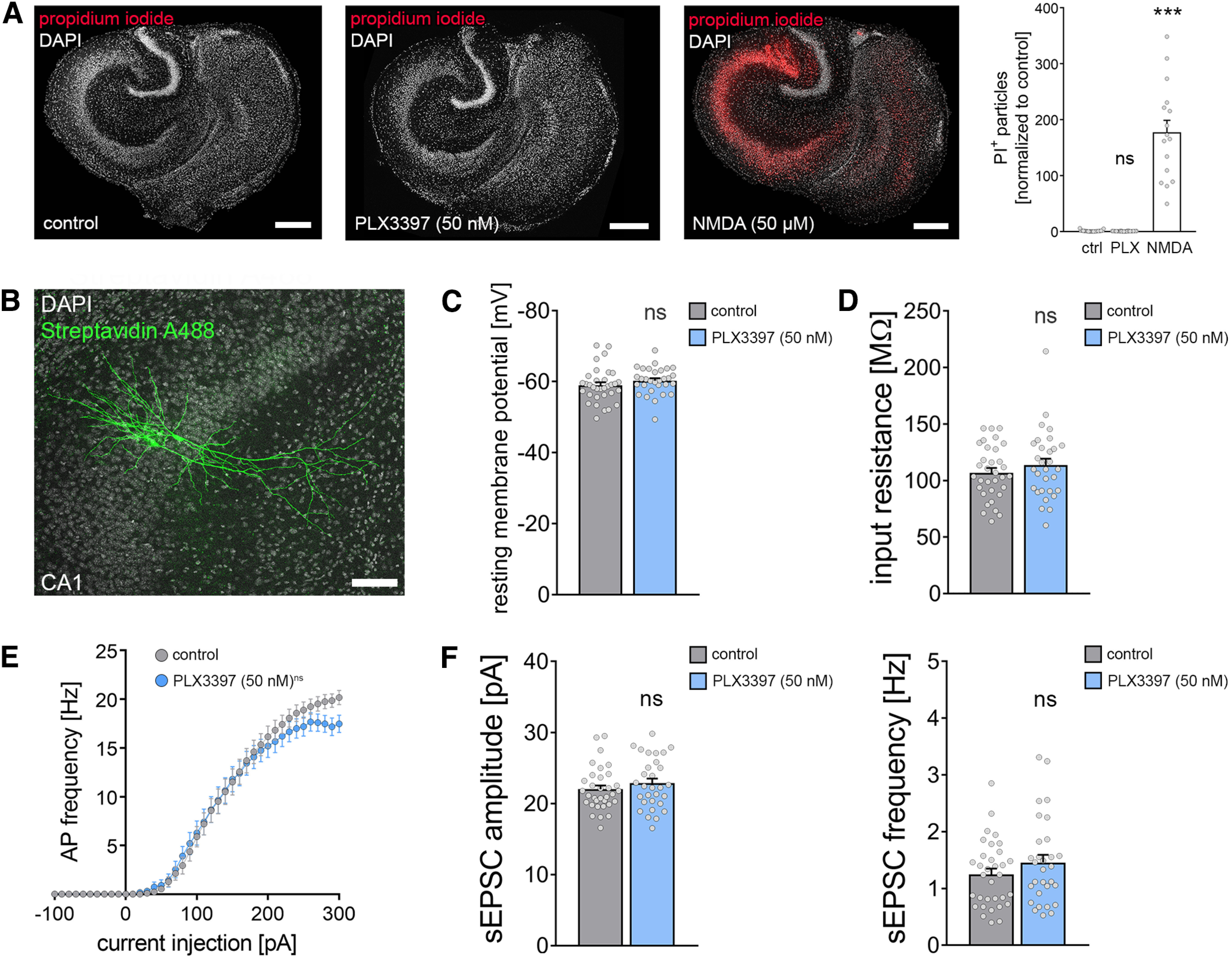
Depletion of microglia does not affect cell viability and basic functional properties of CA1 pyramidal neurons. ***A***, Tissue cultures stained with PI [left: vehicle control, middle: PLX3397 (50 nm, 18 d), right: NMDA (50 μm, 4 h)]. Group data of the quantified PI signals are shown in the graph on the right (n_control_ = 29 cultures, n_PLX(50 nm)_ = 28 cultures, n_NMDA_ = 16 cultures; Kruskal–Wallis test followed by Dunn's *post hoc* correction). Scale bars, 200 µm. ***B***, Examples of patched, recorded, and *post hoc* identified CA1 pyramidal neurons. Scale bar, 100 µm. ***C***, ***D***, Group data of passive membrane properties of CA1 pyramidal neurons in microglia-depleted (i.e., PLX3397; 50 nm, 18 d) treated tissue cultures and control cultures (n_control_ = 33 cells, n_PLX3397_ = 30 cells; Mann–Whitney test). ***E***, Group data of AP frequencies of CA1 pyramidal neurons in the respective groups (n_control_ = 33 cells, n_PLX3397_ = 30 cells, repeated measures two-way ANOVA followed by Sidak's multiple comparisons). ***F***, Group data of AMPAR-mediated sEPSCs recorded from CA1 pyramidal neurons revealed no significant changes in excitatory neurotransmission in microglia-depleted tissue cultures (n_control_ = 33 cells, n_PLX3397_ = 30 cells; Mann–Whitney test). Gray dots represent individual data points. Data are mean ± SEM. ***p < 0.001.

The neurotrophic and neuroprotective effects of microglia are well recognized in the field (e.g., Jin et al., 2017; Freria et al., 2020). Although no obvious signs of cell death were observed in our experiments and despite the fact that baseline mEPSC properties were comparable between the two groups (compare Fig. 3*C*,*F*), we decided to err on the side of caution and tested for alterations in cell viability as a potential confounding factor of rMS-induced synaptic plasticity. In these experiments, PI staining was used to assess viability and cell death in our preparations (Fig. 4) (compare Lenz et al., 2020). PI is a cell membrane-impermeant fluorescent molecule that binds to DNA. Hence, PI can be used as a marker for membrane integrity when applied to living tissue. Tissue cultures treated with PLX3397 (50 nm) or vehicle-only controls were stained with PI, and NMDA-treated tissue cultures (50 μm, 4 h) served as a positive control in these experiments (Fig. 4*A*; Kruskal–Wallis test with Dunn's *post hoc* correction; control-PLX: *p* = 0.91; control-NMDA: *p* < 0.001). The PI signal was comparatively low in 3-week-old control cultures and age- and time-matched PLX3397-treated preparations, while a significant increase in PI signal was detected in the NMDA-treated group (Fig. 4*A*). We conclude from these results that the inability of CA1 pyramidal neurons to express rMS-induced synaptic plasticity is not based on altered cell viability or cell death in the absence of microglia.

### No major functional and structural changes of CA1 pyramidal neurons in microglia-depleted tissue cultures

**Figure 5.**
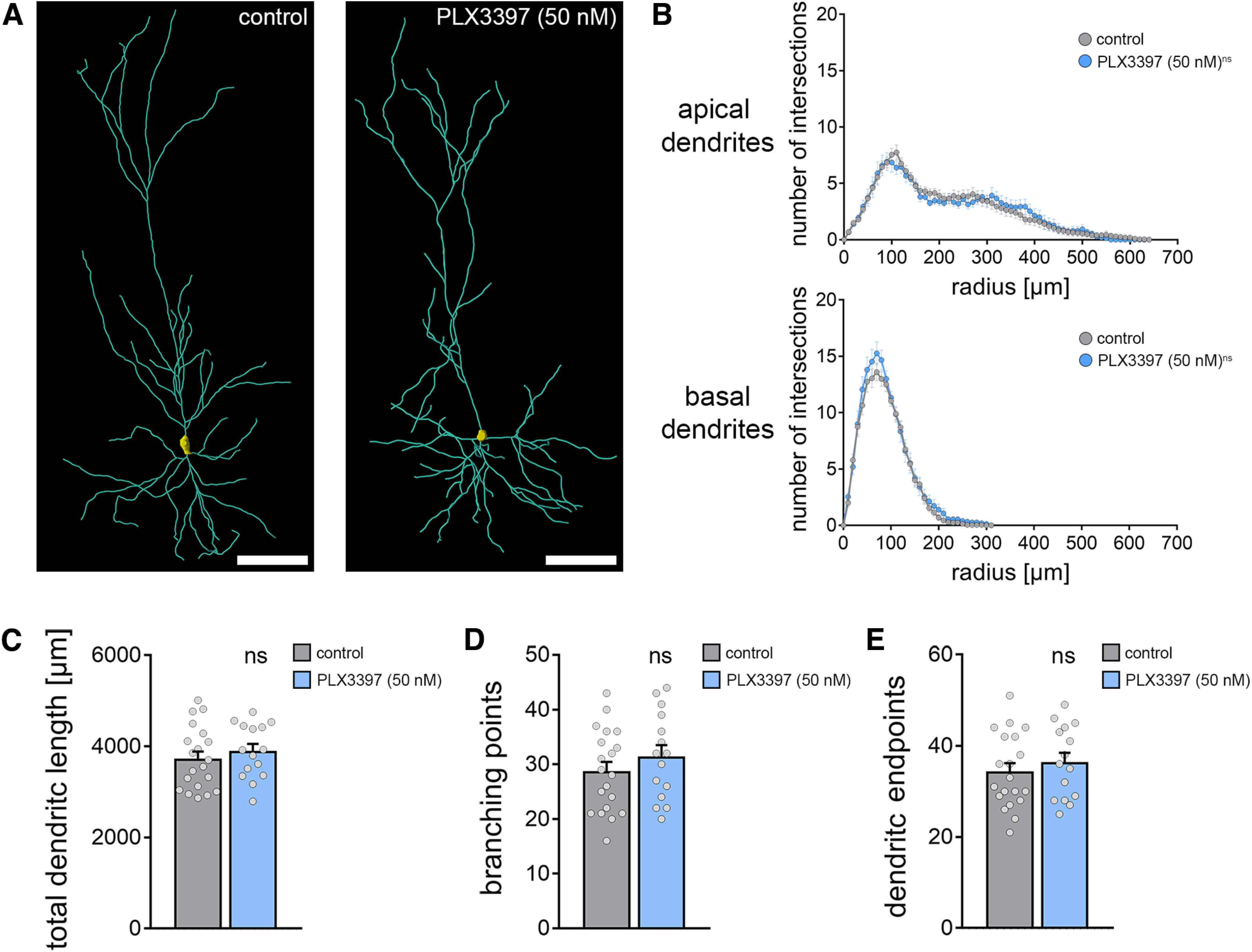
Dendritic morphology of CA1 pyramidal cells is not affected in microglia-depleted tissue cultures. ***A***, Examples of three-dimensionally reconstructed CA1 pyramidal neurons in nondepleted controls and PLX3397-treated (i.e., microglia-depleted tissue cultures). Scale bars, 100 µm. ***B***, Sholl analysis of apical and basal dendrites from reconstructed CA1 neurons in the respective groups (n_control_ = 20 cells, n_PLX3397_ = 15 cells; repeated measures two-way ANOVA followed by Sidak's multiple comparisons). ***C-E***, Group data of additional morphologic parameters from the same set of reconstructed CA1 pyramidal neurons in microglia-depleted tissue cultures and vehicle-only treated control cultures (n_control_ = 20 cells, n_PLX3397_ = 15 cells; Mann–Whitney test). Gray dots represent individual data points. Data are mean ± SEM.

**Figure 6.**
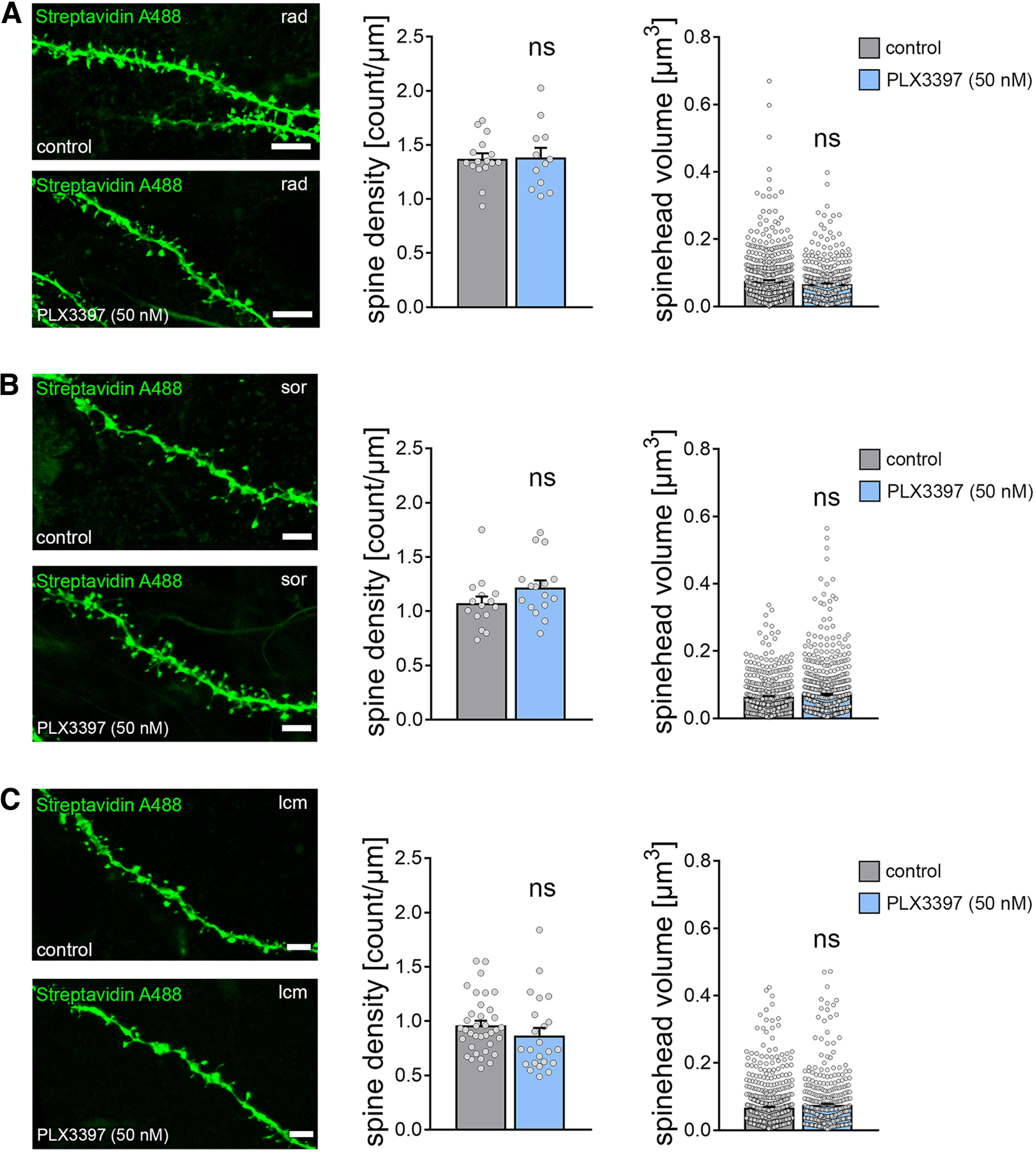
Dendritic spines of CA1 pyramidal neurons are not altered in microglia-depleted tissue cultures. ***A-C***, Examples of dendritic segments and group data of spine densities and spine volumes from patched and *post hoc* identified CA1 pyramidal neurons in stratum radiatum (rad, ***A***), stratum oriens (sor, ***B***), and stratum lacunosum-moleculare (lcm, ***C***) of PLX3397-treated, that is, microglia-depleted tissue cultures and control cultures (rad density: n_control_ = 16 dendritic segments, n_PLX3397_ = 12 dendritic segments; rad volume: n_control_ = 578 spines, n_PLX3397_ = 393 spines; sor density: n_control_ = 15 dendritic segments, n_PLX3397_ = 16 dendritic segments; sor volume: n_control_ = 450 spines, n_PLX3397_ = 703 spines; lcm density: n_control_ = 36 dendritic segments, n_PLX3397_ = 23 dendritic segments; lcm volume: n_control_ = 655 spines, n_PLX3397_ = 405 spines; Mann–Whitney test). Scale bars, 3 µm. Gray dots represent individual data points. Data are mean ± SEM.

Neuronal excitability and morphology are expected to have a major impact on the outcome of electromagnetic stimulation (Aberra et al., 2020; Shirinpour et al., 2021). Therefore, we tested for the effect of microglia depletion on structural and functional properties of CA1 pyramidal neurons in our experiments (Figs. 4–6). Another set of CA1 pyramidal neurons was recorded in control and microglia-depleted tissue cultures and stained for *post hoc* morphologic analysis (Fig. 4*B*; compare Figs. 5 and 6).

PLX3397-mediated microglia depletion did not affect the basic functional properties of CA1 pyramidal neurons (i.e., resting membrane potential) (Fig. 4*C*; Mann–Whitney test, *p* = 0.08, *U* = 366) and input resistance (Fig. 4*D*; Mann–Whitney test, *p* = 0.53, *U* = 449). A slight but nonsignificant difference in AP frequency was observed for high current injections between the two groups (Fig. 4*E*; repeated measures two-way ANOVA with Sidak's multiple comparisons; *p*_treatment_ = 0.12, *F* = 2.44; *p*_current injection_<0.001, *F* = 164.3). Likewise, amplitudes and frequencies of AMPAR-mediated sEPSCs were not significantly different between the groups (Fig. 4*F*, Mann–Whitney test; sEPSC amplitude: *p* = 0.33, *U* = 424; sEPSC frequency: *p* = 0.40, *U* = 433). These results indicate that microglia depletion does not affect basic functional properties and synaptic activity of CA1 pyramidal neurons.

Consistent with these findings, Sholl analysis of reconstructed apical and basal dendrites did not show any major effects of microglia depletion on the dendritic morphologies of CA1 pyramidal neurons (Fig. 5*A*,*B*; repeated-measures two-way ANOVA with Sidak's multiple comparisons; apical dendrites: *p*_treatment_ = 0.90, *F* = 0.02; *p*_radius_<0.001, *F* = 44.55; basal dendrites: *p*_treatment_ = 0.19, *F* = 1.81; p_radius_<0.001, *F* = 187.8). We did not observe any significant differences in total dendritic length (Fig. 5*C*; Mann–Whitney test; *p* = 0.42, *U* = 125), the number of branching points (Fig. 5*D*; Mann–Whitney test; *p* = 0.33, *U* = 120.5), or the number of endpoints (Fig. 5*E*; Mann–Whitney test; *p* = 0.51, *U* = 130).

Finally, a detailed morphologic analysis of CA1 dendritic spines confirmed our sEPSC recordings by demonstrating no significant differences in spine densities and spine-head volumes between the two groups in CA1 stratum radiatum (rad, Fig. 6*A*; Mann–Whitney test; spine density: *p* = 0.84, *U* = 91; spine head volume: *p* = 0.19, *U* = 108006), stratum oriens (sor, Fig. 6*B*; Mann–Whitney test; spine density: *p* = 0.09, *U* = 77; spine head volume: *p* = 0.56, *U* = 154981), and stratum lacunosum-moleculare (lcm, Fig. 6*C*; Mann–Whitney test; spine density: *p* = 0.08, *U* = 300; spine head volume: *p* = 0.31, *U* = 127711).

Together, we conclude that microglia depletion does not cause major structural and functional changes in CA1 pyramidal neurons that could readily explain the inability of neurons to express rMS-induced synaptic plasticity in the absence of microglia.

### Realistic multiscale computer modeling predicts no major differences in rMS-induced depolarization and intracellular Ca^2+^ levels

**Figure 7.**
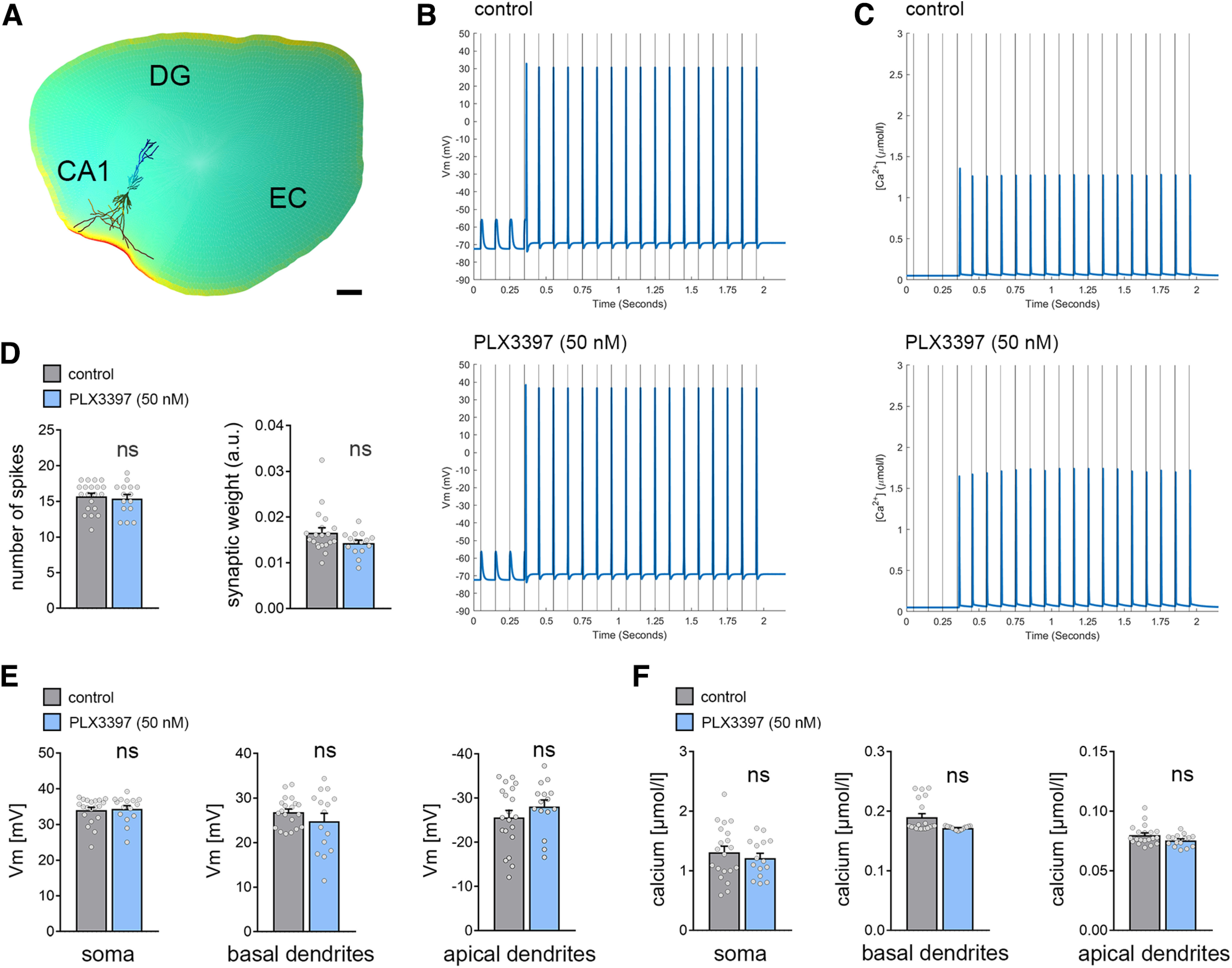
Multiscale computer modeling of rMS. ***A***, Neuronal responses to rMS were modeled in realistic dendritic morphologies from reconstructed CA1 pyramidal neurons in a representative tissue culture environment. EC, entorhinal cortex; DG, dentate gyrus. Scale bar, 200 µm. ***B***, ***C***, Changes in membrane voltage (*V*_m_; ***B***) and intracellular calcium levels (Ca^2+^; ***C***) were assessed for a train of 20 pulses at 10 Hz at the constant stimulation intensity used in the experimental setting. CA1 pyramidal neurons in both conditions showed a delayed suprathreshold response on rMS. ***D***, Both the number of cellular spikes on stimulation and the synaptic weight did not show a significant difference between CA1 pyramidal neurons from microglia-depleted and nondepleted control cultures (n_vehicle-only_ = 20 cells, n_PLX3397_ = 15 cells; GLMMs). ***E***, In the same set of cells, the peak membrane voltage difference in response to magnetic stimulation was modeled in the somatic, apical, and basal dendritic compartments. No differences were observed between the two groups, respectively. ***F***, Analysis of stimulus-triggered changes in intracellular calcium levels. No changes in both the dendritic and the somatic compartments were evident between CA1 pyramidal neurons of microglia-depleted tissue cultures and control cultures. Gray dots represent individual data points. Data are mean ± SEM.

We further evaluated the effects of 10 Hz rMS on CA1 pyramidal neurons using multiscale computational modeling to link the physical input parameters of rMS to dendritic morphologies and subcellular neural effects (Fig. 7) (Shirinpour et al., 2021). The 3D reconstructed morphologies from recorded CA1 pyramidal neurons in microglia-depleted and nondepleted tissue cultures (compare Fig. 5) were used in these experiments (Fig. 7*A*). Both membrane voltage and calcium concentrations were assessed in this modeling approach (Fig. 7*B–F*). We estimated the minimum synaptic weight just below the firing threshold of the CA1 pyramidal neurons. Consistent with our experimental data, the synaptic weights of the model did not differ in CA1 pyramidal neurons from depleted and nondepleted tissue cultures (Fig. 7*D*).

We compared the peak spike values for the membrane voltage. The winning model (ΔBIC = 341.24; *F*_(2102)_ = 1387.59, *p* = 2.2 × 10^−16^) used the compartment as predictor. Confirming our expectations, the analysis revealed significantly weaker peak values in the apical (*t* = −48.524, *p* = 2.2 × 10^−16^) and basal dendrites (*t* = −6.563, *p* = 2.23 × 10^−9^). However, the treatment had no effect on either the number of spikes (Fig. 7*D*) or the peak AP values in any compartment (Fig. 7*E*).

We continued by comparing the peak calcium-concentration values extracted from the compartments and treatment conditions. As for the voltage data, the winning model (ΔBIC = 189.39; *F*_(2102)_ = 283.51, *p* = 2.2 × 10^−16^) used the compartment as predictor. Again, the analysis revealed significantly weaker peak values in the apical (*t* = −21.64, *p* = 2.2 × 10^−16^) and basal dendrites (*t* = −19.75, *p* = 2.2 × 10^−16^). However, the treatment had no major effects on the peak calcium-concentration levels in compartments investigated (Fig. 7*F*).

### rMS does not affect structural properties of microglia

**Figure 8.**
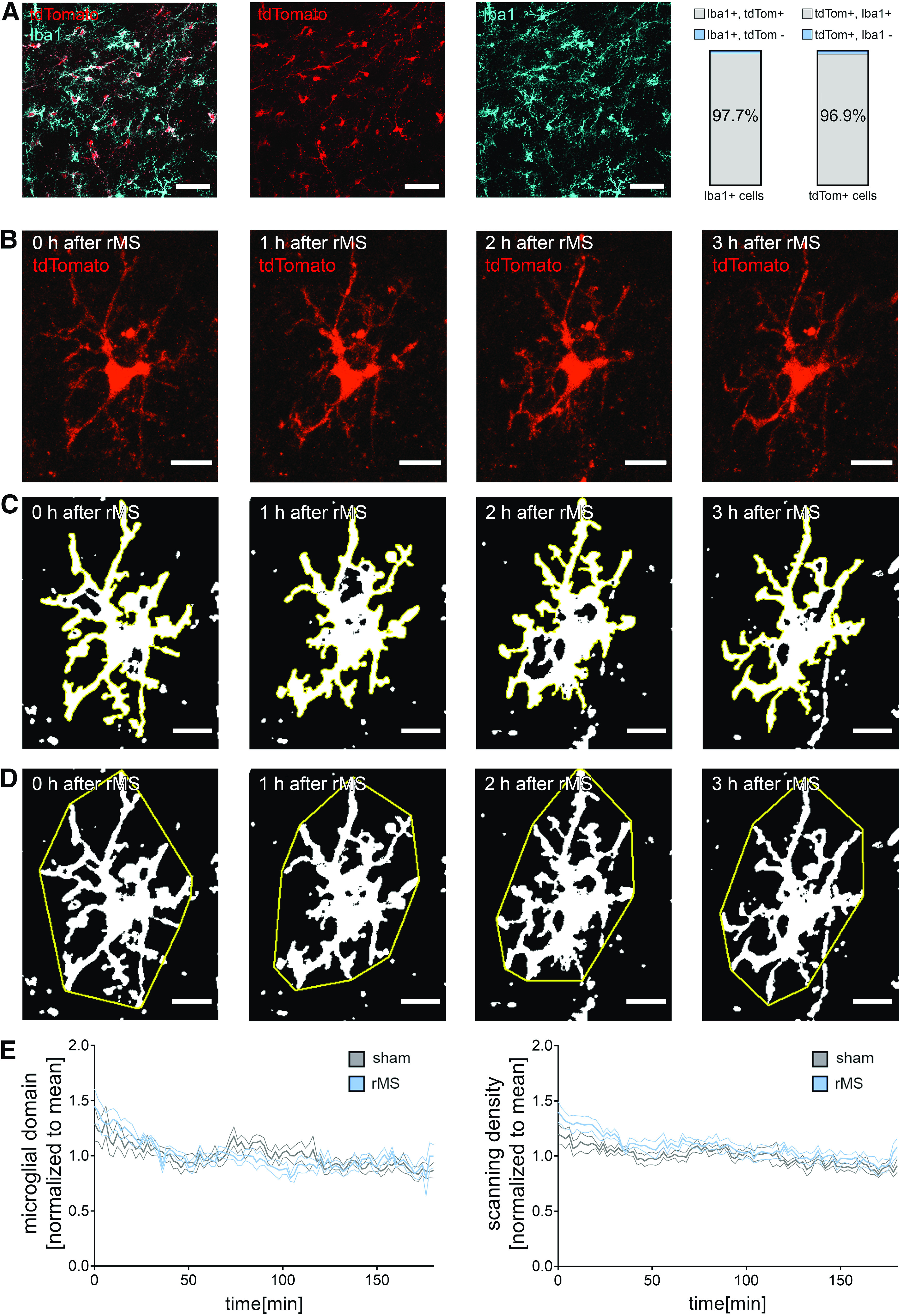
rMS does not affect microglia morphology. ***A***, Representative image of Iba1-stained tissue culture prepared from homozygous *HexB^tdT/tdT^* mice. Note tandem dimer (td) Tomato-expressing microglia. Right, Group data showing an almost complete overlap of the two signals (n_Iba1+_ = 273 cells in 5 cultures; n_tdTomato+_ = 274 cells in 5 cultures.). Scale bars, 50 µm. ***B-D***, Examples of tdTomato-expressing microglia in hippocampal area CA1 imaged from *HexB^tdT/tdT^* cultures over a period of 3 h following 10 Hz rMS (2 min intervals). Maximum intensity projections of image stacks (***B***), microglial scanning densities (***C***), and microglial domains (***D***) are illustrated. Scale bars, 15 µm. ***E***, Group data of microglial domains and scanning densities from rMS-stimulated and sham-stimulated tissue cultures (n_sham_ = 6 cells, n_rMS_ = 6 cells from 6 cultures in each group; repeated measures two-way ANOVA followed by Sidak's multiple comparisons). Data are mean ± SEM.

**Figure 9.**
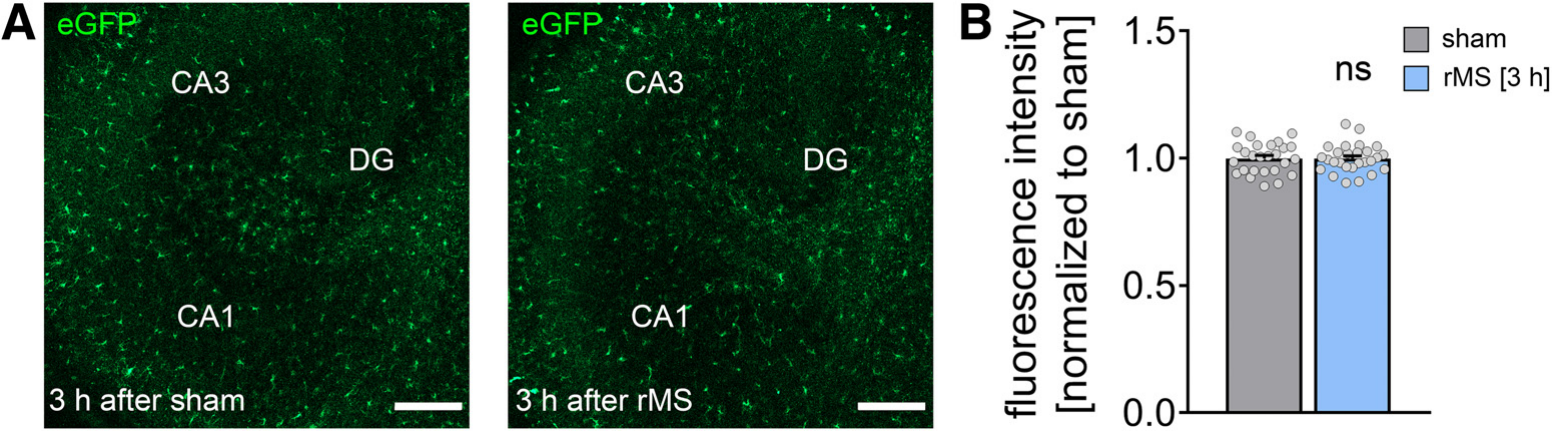
rMS does not induce neuroinflammation. Sample images (***A***) and group data (***B***) of eGFP fluorescence intensity of rMS-stimulated and sham-stimulated tissue cultures prepared from TNF-reporter mice [*C57BL/6-Tg(TNFa-eGFP)*] imaged 3 h after 10 Hz rMS. DG, dentate gyrus. n_sham_ = 26 cultures, n_rMS_ = 27 cultures; Mann–Whitney test. Scale bars, 200 µm. Gray dots represent individual data points. Data are mean ± SEM.

**Figure 10.**
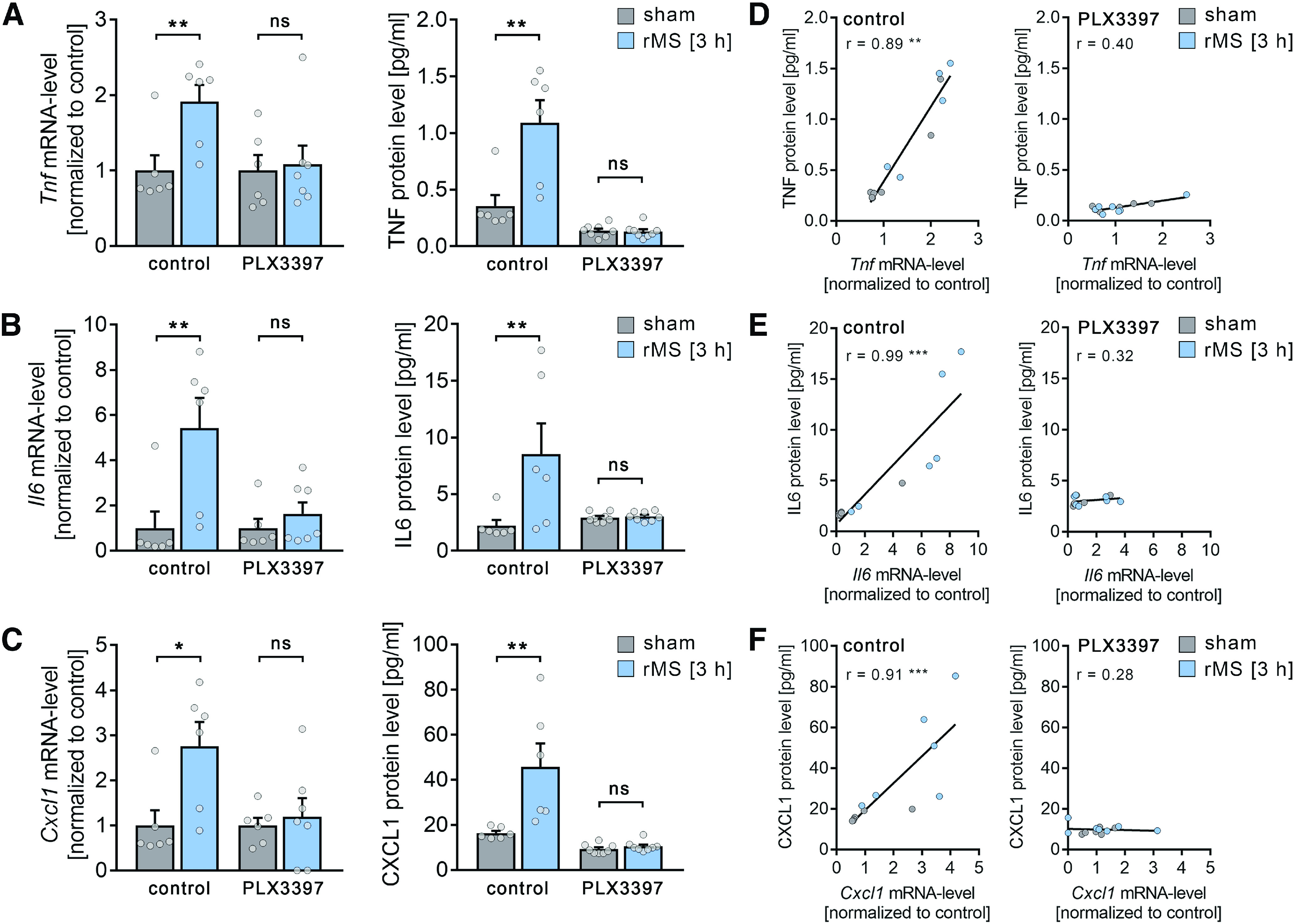
rMS triggers the expression and release of plasticity-promoting microglial factors. ***A-C***, Left panels, group data of mRNA levels of (***A***) *TNF*α, (***B***) *IL6*, and (***C***) *CXCL1* in nondepleted cultures and microglia-depleted cultures (PLX3397-treated cultures) 3 h after 10 Hz rMS and sham stimulation (n_control mRNA_ = 6 cultures, respectively, for each experimental condition, n_PLX3397 mRNA sham_ = 6 cultures, n_PLX3397 mRNA rMS_ = 7 cultures; Mann–Whitney test, U_control Tnf mRNA_ = 2, U_control Il6 mRNA_ = 2, U_control Cxcl1 mRNA_ = 3; for detailed statistical assessment, see Extended Data Table 10-1). Right panels, group data of protein levels of (***A***) TNFα, (***B***) IL6, and (***C***) CXCL1 in culturing medium of nondepleted cultures and microglia-depleted cultures (PLX3397-treated cultures) 3 h after 10 Hz rMS and sham stimulation (n_control protein_ = 6 culturing medium samples, respectively, for each experimental condition, n _PLX3397 protein_ = 8 culturing medium samples, respectively, for each experimental condition; Mann–Whitney test, U_control TNF protein_ = 2, U_control IL6 protein_ = 2, U_control CXCL1 protein_ = 0; for detailed statistical assessment, see Extended Data Table 10-1). ***D-F***, Correlation of mRNA levels and protein levels of TNFα (***D***), IL6 (***E***), and CXCL1 (***F***) in nondepleted control cultures (left panels) and microglia-depleted (PLX3397-treated, right panels) cultures 3 h after 10 Hz rMS and sham stimulation (n_control_ = 6 cultures or culturing medium samples, respectively, for each experimental condition, n_PLX3397-sham_ = 6 cultures or culturing medium samples, n_PLX3397-rMS_ = 7 cultures or culturing medium samples; for detailed statistical assessment, see Extended Data Table 10-2). Gray dots represent individual data points. Data are mean ± SEM. *p < 0.05. **p < 0.01. ***p < 0.001.

After demonstrating that structural and functional differences of CA1 pyramidal neurons do not explain our major findings (i.e., the inability of neurons to express rMS-induced synaptic plasticity in the absence of microglia), we considered the possible effects of rMS on the structural and functional properties of microglia (Figs. 8–10).

10.1523/JNEUROSCI.2226-22.2023.tab10-1 Table 10-1 : Statistical assessment of mRNA (qRT-PCR) and protein detection (MSD assay) results. Download Table 10-1, XLSX file.

10.1523/JNEUROSCI.2226-22.2023.tab10-2 Table 10-2 : Linear regression analysis of mRNA and protein levels from cytokine detection assays. Download Table 10-2, XLSX file.

To test for the effects of 10 Hz rMS on microglia morphology and dynamics, tissue cultures were prepared from *HexB-tdTom* mice, which express the red fluorescent protein tandem dimer (td) Tomato under the control of the Hexoaminidase B promotor (HexB) (Masuda et al., 2020). Immunostainings of tissue cultures confirmed that 97% of tdTomato-positive cells also expressed Iba1, and in 98% of all Iba1-positive cells tdTomato signal was detected (Fig. 8*A*). Apparently, the vast majority of microglia are readily identified by tdTomato in 3-week-old tissue cultures prepared from *HexB-tdTom* mice.

Live-cell microscopy was used to image microglia (i.e., tdTomato-expressing cells in CA1 stratum radiatum) (Fig. 8*B–E*). Confocal image stacks were obtained every 2 min for 3 h immediately after 10 Hz rMS or sham stimulation (Fig. 8*B*). In these experiments, we did not observe any major rMS-related changes in microglia morphology. A detailed analysis of microglia dynamics in maximum-intensity projections (Fig. 8*B–D*) revealed no significant changes in dynamic microglial domains and scanning densities among rMS-stimulated and sham-stimulated tissue cultures (Fig. 8*E*; repeated-measures two-way ANOVA with Sidak's multiple comparisons; microglial domain: *p*_stimulation_ = 0.34, *F* = 1.0; *p*_time_<0.001, *F* = 4.95; scanning density: *p*_stimulation_ = 0.02; no significant difference detected in multiple comparisons, *F* = 7.87; *p*_time_<0.001, *F* = 6.73). We conclude that 10 Hz rMS does not lead to major changes in microglia morphology and dynamics.

We further excluded an rMS-induced pathologic activation of microglia in tissue cultures prepared from a transgenic TNFα reporter mouse line, which expresses the eGFP under the control of the TNF promoter (Fig. 9*A*, *C57BL/6-Tg(TNFa-eGFP)*). In a previous study, we found a strong pathologic activation of microglia (i.e., strong increase in eGFP expression) in the presence of bacterial lipopolysaccharides (Lenz et al., 2020). Here, live-cell microscopy revealed no obvious changes in eGFP fluorescence following 10 Hz rMS (Fig. 9*B*; Mann–Whitney test, *p* = 0.91, *U* = 344). We conclude that 10 Hz rMS does not cause an inflammatory response in organotypic tissue cultures.

### rMS triggers an activity-dependent release of plasticity-promoting cytokines from microglia

The plasticity-modulating effects of cytokines secreted by microglia under physiological conditions (i.e., in the absence of major changes in microglia morphology and inflammation) are well established (Tancredi et al., 2000; Stellwagen and Malenka, 2006; Santello et al., 2011; Cao et al., 2014; Habbas et al., 2015; Maggio and Vlachos, 2018; Chai et al., 2019; Heir and Stellwagen, 2020; Lenz et al., 2020). Specifically, TNFα and IL6 have been linked to the ability of neurons to express synaptic plasticity (Tancredi et al., 2000; Stellwagen and Malenka, 2006; Rizzo et al., 2018). In this context, we were recently able to demonstrate that low concentrations of TNFα promote the ability of neurons to express synaptic plasticity (Maggio and Vlachos, 2018). Therefore, we theorized that 10 Hz rMS could trigger an activity-dependent production and/or secretion of TNFα and other cytokines at low plasticity-promoting concentrations.

We performed qPCR-analyses and protein-detection assays in 10 Hz stimulated and sham-stimulated microglia-depleted and nondepleted tissue cultures (Fig. 10). Indeed, 10 Hz rMS triggered an increase in TNFα (Fig. 10*A*), IL6 (Fig. 10*B*), and chemokine ligand 1 (CXCL1) (Fig. 10*C*) that was absent in microglia-depleted tissue cultures (detailed statistics provided in Extended Data Table 10-1). A positive correlation of protein levels and mRNA levels was evident for TNFα (Fig. 10*D*), IL6 (Fig. 10*E*), and CXCL1 (Fig. 10*F*) in the nondepleted tissue cultures (detailed statistics provided in Extended Data Table 10-2).

**Figure 11.**
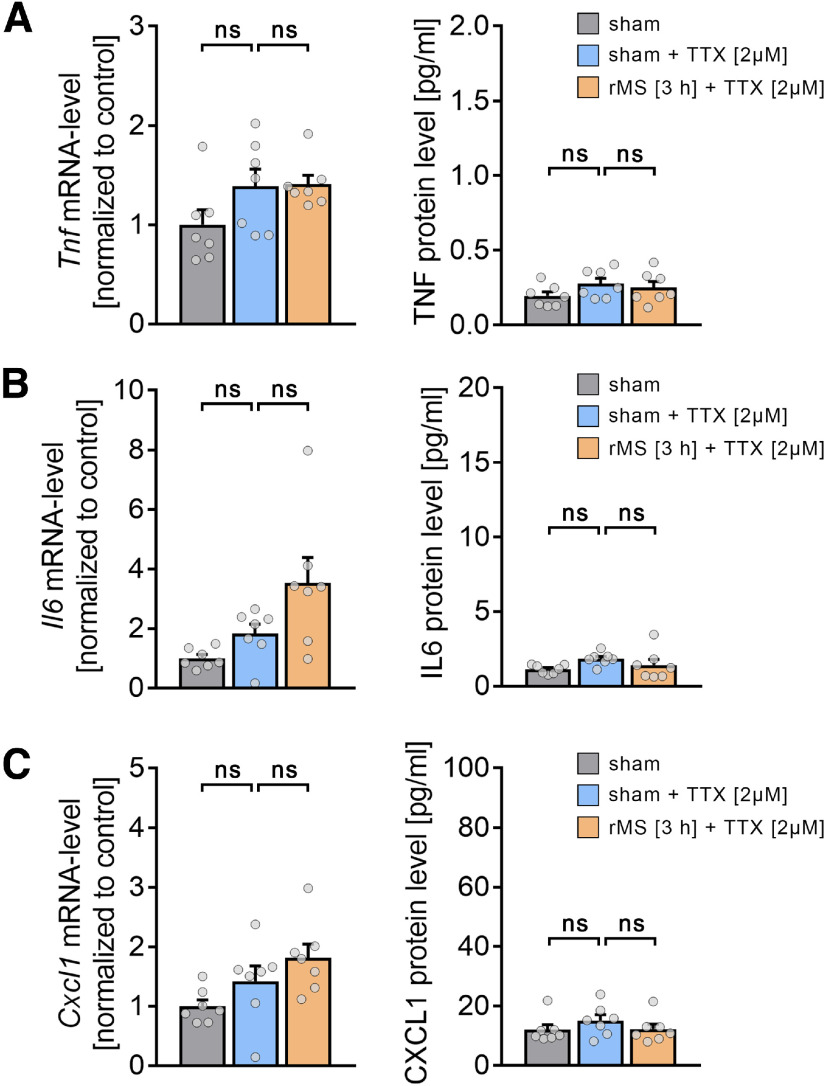
Activity-dependent release of microglial cytokines. ***A-C***, Group data of mRNA levels (right panels) and protein levels in the incubation medium (left panels) of (***A***) TNFα, (***B***) IL6, and (***C***) CXCL1 in nondepleted cultures 3 h after sham stimulation, sham stimulation in TTX (2 μm), and 10 Hz rMS in TTX (2 μm; *n* = 7 cultures, respectively, for each experimental condition; Kruskal–Wallis test followed by Dunn's *post hoc* correction; for detailed statistical assessment, see Extended Data Table 11-1). Gray dots represent individual data points. Data are mean ± SEM.

In order to test whether the rMS-induced release of cytokines from microglia is activity-dependent, in a different set of nondepleted tissue cultures, we blocked voltage-gated sodium channels with 2 μm TTX during the stimulation (Fig. 11). Indeed, no significant changes in TNFα (Fig. 11*A*), IL6 (Fig. 11*B*), and CXCL1 (Fig. 11*C*), both at mRNA and protein levels, were observed in these experiments (Kruskal–Wallis test; detailed statistics provided in Extended Data Table 11-1). These results show that 10 Hz rMS induces an activity-dependent release of microglial cytokines.

10.1523/JNEUROSCI.2226-22.2023.tab11-1 Table 11-1 : Statistical assessment of cytokine detection assays from repetitive magnetic stimulation in the presence of TTX. Download Table 11-1, XLSX file.

### Substitution of plasticity-promoting cytokines during stimulation rescues rMS-induced synaptic plasticity in microglia-depleted tissue cultures

**Figure 12.**
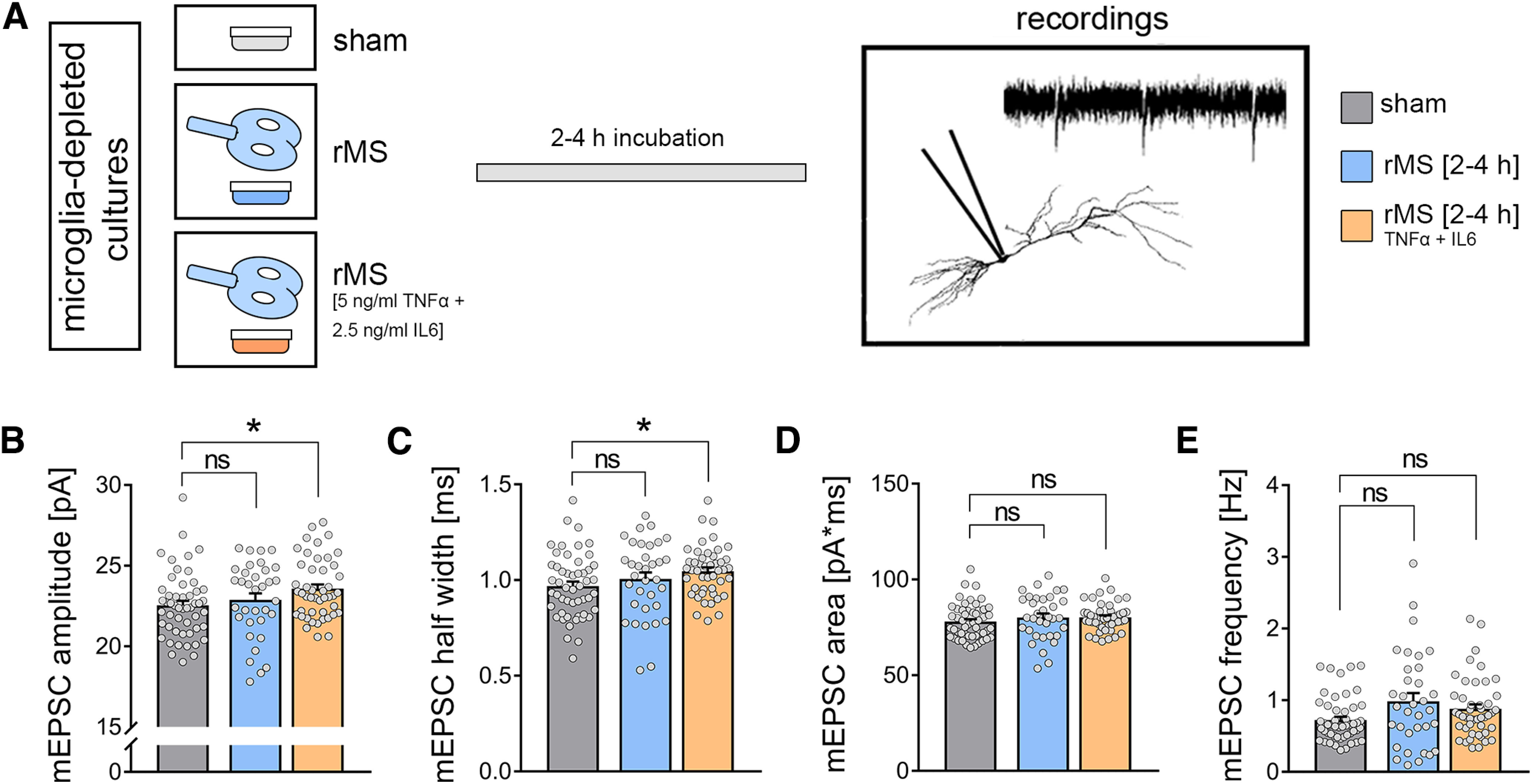
Substitution of pro-inflammatory cytokines during stimulation in microglia-depleted tissue cultures rescues rMS-induced synaptic plasticity. ***A***, Schematic of the experimental design. A subset of tissue cultures was stimulated in the presence of TNFα (5 ng/ml) and IL6 (2.5 ng/ml). Effects of TNFα and IL6 in sham-stimulated cultures are reported in the main text. ***B***-***E***, Group data of AMPAR-mediated mEPSCs recorded from CA1 pyramidal neurons in sham-stimulated and 10 Hz rMS-stimulated cultures (n_sham_ = 51 cells, n_rMS_ = 34 cells, n_rMS-TNF+IL6_ = 46 cells; Kruskal–Wallis test followed by Dunn's *post hoc* correction). Gray dots represent individual data points. Data are mean ± SEM. *p < 0.05.

We then tested whether the exogenous substitution of TNFα and IL6 rescues the ability of CA1 pyramidal neurons to express rMS-induced synaptic plasticity in the absence of microglia (Fig. 12). TNFα (5 ng/ml) and IL6 (2.5 ng/ml) were added to the medium during stimulation only, and AMPAR-mediated mEPSCs were recorded from CA1 pyramidal neurons of microglia-depleted tissue cultures between 2 and 4 h after stimulation (Fig. 12*A*). Indeed, in these experiments, rMS-induced synaptic strengthening, that is, a significant increase in mean mEPSC amplitude (Fig. 12*B*; Kruskal–Wallis test, sham-rMS: *p* = 0.31, sham-rMS+cytokines: *p* = 0.03) and half-width (Fig. 12*C*; Kruskal–Wallis test, sham-rMS: *p* = 0.32, sham-rMS+cytokines: *p* = 0.03), was observed while mEPSC area (Fig. 12*D*; Kruskal–Wallis test, sham-rMS: *p* = 0.38, sham-rMS+cytokines: *p* = 0.35) and mEPSC frequency (Fig. 12*E*; Kruskal–Wallis test, sham-rMS: *p* = 0.11, sham-rMS+cytokines: *p* = 0.12) remained unchanged. Of note, exposing microglia-depleted cultures to the same concentrations of TNFα and IL6 under sham conditions did not affect mEPSC amplitudes compared with sham-stimulated microglia-depleted cultures that were not exposed to TNFα and IL6; only mEPSC frequencies were increased in tissue cultures exposed only to TNFα and IL6 (n_sham_ = 20 cells, n_sham+cytokines_ = 22 cells; mEPSC amplitude: 24.27 ± 0.56 pA (sham) vs 24.86 ± 0.43 pA (sham+cytokines), not significant; mEPSC frequency: 0.81 ± 0.06 Hz (sham) vs 0.95 ± 0.05 Hz (sham+cytokines), *p < 0.05; Mann–Whitney test). We conclude that TNFα and IL6 released from microglia during stimulation is required for rMS-induced synaptic potentiation.

### *In vivo* microglia depletion prevents rTMS-induced excitatory synaptic plasticity in the mouse mPFC

**Figure 13.**
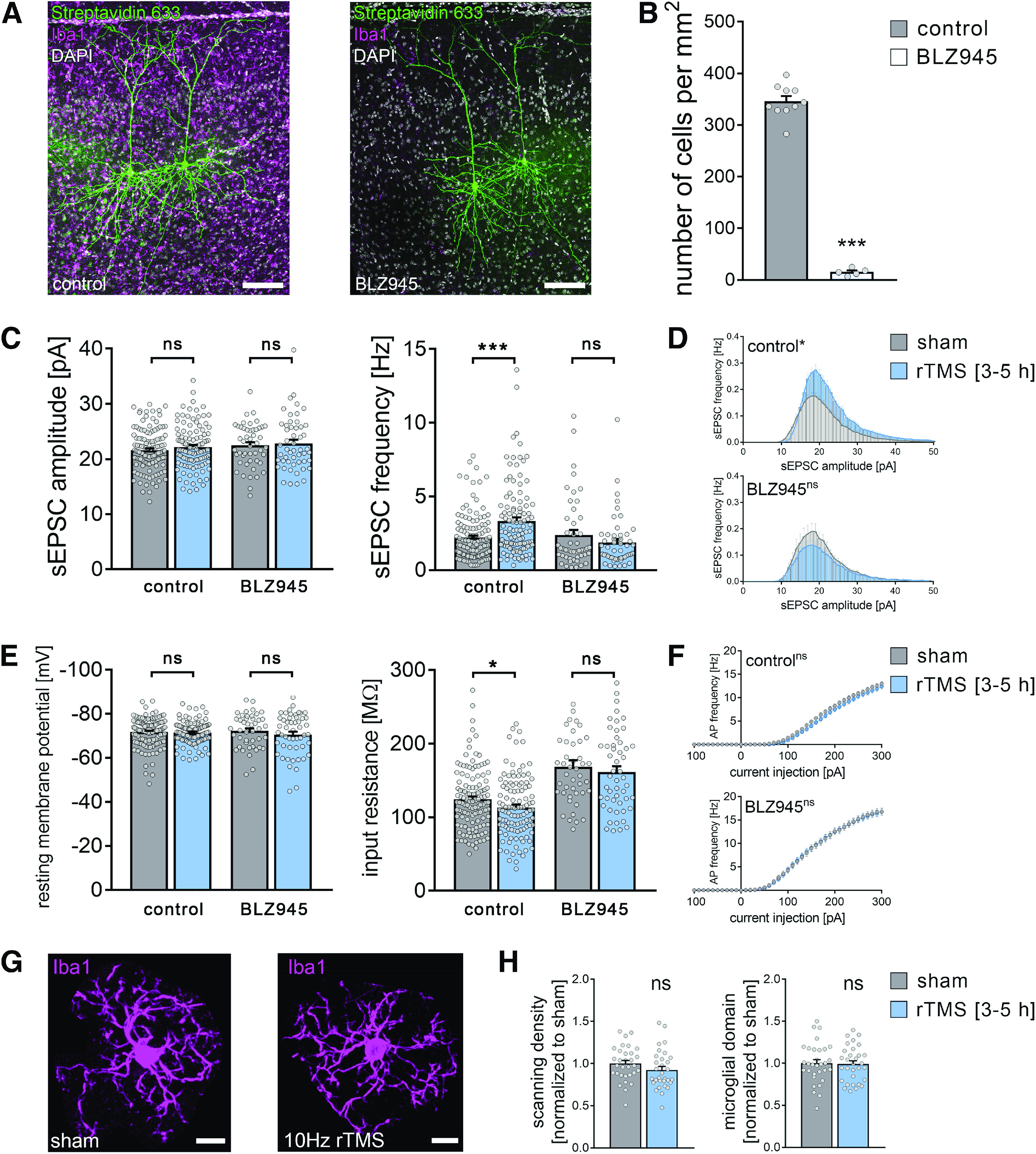
Microglia depletion *in vivo* prevents rTMS-induced synaptic plasticity of superficial pyramidal neurons in the mPFC of adult mice. ***A***, *Post hoc* visualization of superficial pyramidal neurons by streptavidin staining in the mPFC of nondepleted (left) and microglia-depleted (BLZ945-treated, right) adult mice. Microglia visualized by Iba1 immunolabeling. Scale bars, 100 µm. ***B***, Quantification of microglia numbers confirmed an ∼90% reduction of microglia in the mPFC of BLZ945-treated animals (n_control_ = 10 animals, n_microglia-depleted (BLZ945)_ = 5 animals; Mann–Whitney test, *U* = 0). ***C***, Group data of AMPAR-mediated sEPSCs recorded from layer 2/3 pyramidal neurons in the mPFC of nondepleted and microglia-depleted mice 3-5 h after 10 Hz rTMS and sham stimulation (nondepleted animals: n_sham_ = 124 cells from 8 animals, n_rTMS_ = 111 cells from 8 animals; microglia-depleted [BLZ945-treated] animals: n_sham_ = 48 cells from 2 animals, n_rTMS_ = 51 cells from 3 animals; Mann–Whitney test, U_sEPSC frequency, nondepleted_ = 2324). ***D***, Amplitude-frequency plots of sEPSC recordings from the respective groups (repeated measures two-way ANOVA following Sidak's multiple comparisons test). ***E***, ***F***, Group data of passive and active membrane properties recorded from layer 2/3 pyramidal neurons in the mPFC of nondepleted and microglia-depleted mice 3-5 h after 10 Hz rTMS and sham stimulation (n_control-sham_ = 124 cells, n_control-rTMS_ = 111 cells, n_BLZ-sham_ = 44 cells, n_BLZ-rTMS_ = 51 cells; Mann–Whitney test and repeated measures two-way ANOVA followed by Sidak's multiple comparisons test for AP frequency analysis; U_control-input resistance_ = 5836). One data point (input resistance_BLZ-sham_ = 413.8 mΩ) is outside the *y* axis limits. ***G***, ***H***, Representative images and group data of Iba1-stained microglia in the mPFC of nondepleted mice 3-5 h after 10 Hz rTMS and sham stimulation (n_sham_ = 31 cells from 5 animals, n_rTMS_ = 31 cells from 5 animals; Mann–Whitney test). Scale bars, 10 µm. Gray dots represent individual data points. Data are mean ± SEM. *p < 0.05. ***p < 0.001.

Finally, we tested whether the presence of microglia is required for rTMS-induced plasticity of synaptic transmission *in vivo* (Thimm and Funke, 2015; Lenz et al., 2016; Kloosterboer and Funke, 2019). *In vivo* microglia depletion was achieved through daily application of the CSF-1R inhibitor BLZ945 (200 mg kg^−1^, oral gavage, for 7 consecutive days before experimental assessment) (Hagemeyer et al., 2017; Masuda et al., 2020). Anesthetized microglia-depleted and nondepleted mice were stimulated with the same 10 Hz stimulation protocol as in our *in vitro* experiments (compare Lenz et al., 2016). Two hours after the stimulation, frontal brain sections containing the mPFC were prepared and AMPAR-meditated sEPSCs were recorded from superficial pyramidal neurons of the mPFC (3-5 h after stimulation; Fig. 13*A*). BLZ945-induced microglia depletion was confirmed by Iba1 stainings of frontal brain sections (∼90% reduction in cell density in the BLZ945-treated animals; Fig. 13*B*; Mann–Whitney test; *p* < 0.01, *U* = 0). While *in vivo* microglia depletion had no significant effects on baseline synaptic transmission, a significant increase in sEPSC frequencies was observed in nondepleted animals but was absent in microglia-depleted animals. Notably, the mean sEPSC amplitude remained unaltered in microglia-depleted and nondepleted animals (Fig. 13*C*; Mann–Whitney test; sEPSC amplitude: *p*_control_ = 0.35, *U*_control_ = 6395; *p*_BLZ_>0.99, *U*_BLZ_ = 1224; sEPSC frequency: *p*_control_ < 0.001, *U*_control_ = 4729; *p*_BLZ_ = 0.42, *U*_BLZ_ = 1109). Amplitude-frequency plots confirmed that rTMS promoted an amplitude-independent increase in sEPSC frequencies which was absent, if not reversed, in the mPFC of microglia-depleted animals (Fig. 13*C*,*D*; repeated measures two-way ANOVA with Sidak's multiple comparisons; control: *p*_stimulation_<0.001, *F* = 17.73; *p*_sEPSC amplitude bins_<0.001, *F* = 227.9; BLZ945: *p*_stimulation_ = 0.22, *F* = 1.52; *p*_sEPSC amplitude bins_< 0.001, *F* = 55.98).

Despite a small but significant decrease in input resistance on rTMS in nondepleted animals, these changes occurred in the absence of major changes in passive (Fig. 13*E*; Mann–Whitney test; resting membrane potential: *p*_control_ = 0.29, *U*_control_ = 6334; *p*_BLZ_ = 0.56, *U*_BLZ_ = 1044; input resistance: *p*_control_ = 0.04, *U*_control_ = 5836; *p*_BLZ_ = 0.56, *U*_BLZ_ = 1043) and active (Fig. 13*F*; repeated-measures two-way ANOVA with Sidak's multiple comparisons; control: *p*_stimulation_ = 0.12, *F* = 2; *p*_current injection_<0.001, *F* = 567.7; BLZ945: *p*_stimulation_ = 0.94, *F* < 0.01; *p*_current injection_<0.001, *F* = 217.2) membrane properties in microglia-depleted and nondepleted animals.

Similar to our *in vitro* experiments in hippocampal area CA1, no rTMS-induced changes in microglia morphologies were observed in the mPFC of anesthetized mice (Fig. 13*G*,*H*; Mann–Whitney test; *p*_scanning density_ = 0.10, *U*_scanning density_ = 362; *p*_microglial domain_>0.99, *U*_microglial domain_ = 480). These results demonstrate the essential (i.e., plasticity-promoting) role of microglia for rTMS-induced changes in synaptic transmission *in vivo*.

## Discussion

The experiments of this study demonstrate that the presence of microglia is required for the induction of synaptic plasticity triggered by 10 Hz rTMS in brain tissue of mice (*in vitro* and *in vivo*). In this context, a functional activation of microglia was observed as reflected by the activity-dependent gene expression and release of cytokines, while microglial morphologies and dynamics remained unaffected. Indeed, substitution of TNFα and IL6 rescued the ability of neurons to express synaptic plasticity in microglia-depleted brain tissue cultures. These results demonstrate that rTMS effectively modulates microglia function (i.e., cytokine release) in stimulated brain regions, thereby identifying a novel mechanism of action which may explain some of the beneficial effects of rTMS in healthy individuals and patients.

It has been proposed that rTMS-induced after-effects are mediated by “LTP-like” plasticity mechanisms (Ziemann, 2004). This evidence is based on physiological characteristics and pharmacological analogies between studies performed at the system level in human subjects (Korchounov and Ziemann, 2011; Ziemann et al., 2015; Brown et al., 2020) and data obtained from animal models (Hoogendam et al., 2010; Vlachos et al., 2012). Specifically, the effects of pharmacological modulation of NMDARs have been interpreted as evidence for “LTP-like” plasticity, considering the relevance of NMDARs in LTP induction (Vlachos et al., 2012; Lenz et al., 2015; Brown et al., 2020). Our own past work demonstrated that pharmacological inhibition of network activity, NMDARs, or L-type voltage-gated calcium channels blocks the ability of neurons to express synaptic plasticity induced by 10 Hz rMS (Vlachos et al., 2012; Lenz et al., 2015). Consistent with these findings, stimulation in Ca^2+^-free external solution failed to induce synaptic plasticity, thus confirming the relevance of Ca^2+^-dependent signaling pathways in rMS-induced synaptic plasticity (Lenz et al., 2015). Notably, NMDARs and L-type voltage-gated calcium channels are also expressed on microglia (Murugan et al., 2011; Hopp, 2021). Indeed, robust experimental evidence exists demonstrating that the modulation of intracellular Ca^2+^ levels regulates important microglia functions (Laprell et al., 2021). Hence, some of the results obtained in animal models and human studies could be explained, at least in part, by the modulation of calcium signaling pathways in neurons and in microglia. In line with this suggestion, in this study, we provided evidence for an activity-dependent expression and release of plasticity-promoting cytokines on 10 Hz rMS. Pharmacological inhibition of sodium channels with TTX blocked cytokine production and release. Moreover, the detrimental effects of microglia depletion on rMS-induced synaptic plasticity were rescued when the (activity-dependent) cytokines TNFα and IL6 were substituted during stimulation. Additional research is required to clarify whether rTMS acts on microglia directly or whether microglia are modulated indirectly by sensing rTMS-induced changes in neuronal activity, for example, via microglial glutamate receptors, purinergic receptors, or other signaling pathways (Cserep et al., 2020).

Regardless of these considerations, the present study demonstrates that the presence of microglia is required for rTMS-induced synaptic plasticity: CA1 pyramidal neurons in microglia-depleted tissue cultures as well as superficial pyramidal neurons in the mPFC of adult mice did not express rTMS-induced excitatory synaptic plasticity. These findings support the recently emphasized impact of glial cells on the neural effects of noninvasive brain stimulation (Gellner et al., 2021). Interestingly, we did not observe any obvious signs of functional and structural alterations of CA1 pyramidal neurons in microglia-depleted tissue cultures. Consistent with these findings, multiscale compartmental modeling confirmed that basic morphologic and functional properties of neurons do not explain the inability of CA1 pyramidal neurons to express rMS-induced plasticity. We must concede, however, that detailed morphologic reconstructions of axons were not obtained, as it is difficult to reliably visualize and reconstruct the entire axon morphology of individual neurons. Indeed, computational studies emphasize the relevance of axons and myelination in rTMS-induced synaptic plasticity (Fields, 2005; Wang et al., 2018; Aberra et al., 2020; Shirinpour et al., 2021). It should be noted, though, that we did not observe any differences in network activity in our experimental setting, as neither sEPSC amplitudes nor frequencies were affected in microglia-depleted tissue cultures. Also, we did not observe any differences in dendritic spine counts, and no evidence for alterations in oligodendrocyte markers was obtained in our RNA microarray analysis of microglia-depleted tissue cultures. It is thus unlikely that major changes in the structural and functional properties of CA1 pyramidal neurons explain our results, especially considering our rescue experiments in which only short exposure to TNFα and IL6 during stimulation was sufficient to restore rMS-induced synaptic plasticity in the absence of microglia. It should be noted, however, that the effect of 10 Hz rMS in microglia-depleted cultures was not rescued completely (i.e., to the level of nondepleted stimulated cultures) by TNFα and IL6 during the stimulation. While it is difficult to determine the exact concentrations of TNFα and IL6 at synaptic sites, it is likely that additional microglial mechanisms (e.g., signals mediated during physical contacts between microglia and synapses) may contribute to rTMS-induced synaptic plasticity.

Previous studies have demonstrated that proinflammatory cytokines influence excitatory neurotransmission (Stellwagen and Malenka, 2006; Cao et al., 2014; Strehl et al., 2014; Riazi et al., 2015; Maggio and Vlachos, 2018; Sheppard et al., 2019). Specifically, TNFα and IL6 have been implicated as important secreted factors that modulate synaptic transmission (Furukawa and Mattson, 1998; Stellwagen et al., 2005; Garcia-Oscos et al., 2012) and plasticity (Heir and Stellwagen, 2020). In this context, it has been shown that TNFα acts as a permissive factor (Steinmetz and Turrigiano, 2010; Becker et al., 2013), where TNFα per se does not trigger major changes in postsynaptic strength; rather, it modulates the ability of neurons to express plasticity without affecting baseline synaptic transmission, in addition to changes in presynaptic glutamate release (Santello et al., 2011) as also reflected by the increased mEPSC frequencies seen in our experiments, in which microglia-depleted tissue cultures were exposed to TNFα and IL6 without electromagnetic stimulation. Indeed, experiments using classic tetanic electric stimulation showed that low concentrations of TNFα rapidly promote LTP-induction, while high concentrations of TNFα impede the ability of neurons to express LTP, without affecting baseline synaptic transmission (Maggio and Vlachos, 2018). The results align with TNFα's metaplastic effects. In line with this suggestion, pathologic activation of microglia with bacterial lipopolysaccharides, which triggers strong TNFα production (i.e., ∼10-15 fold increase in TNFα-mRNA and ∼2000 fold increase in TNFα protein levels) occludes the ability of CA1 pyramidal neurons to express 10 Hz rMS-induced synaptic plasticity (Lenz et al., 2020). Together with the results of the present study, these findings demonstrate that microglia have an important role in rTMS-induced synaptic plasticity. This calls for a systematic assessment of rTMS-induced microglia plasticity, and raises the intriguing possibility that rTMS recruits metaplasticity by activating microglia. Hence, some of the beneficial effects of rTMS seen in patients may reside, at least in part, in the effects of rTMS on microglia function, which also seem to be involved in promoting the ability of neurons to express “LTP-like” plasticity shortly after stimulation.
