# Supplementary material for: Microglial Cytokines Mediate Plasticity Induced by 10 Hz Repetitive Magnetic Stimulation
Source: J Neurosci. 2023 Apr 26;43(17):3042–60. doi: 10.1523/JNEUROSCI.2226-22.2023 (PMC10146500; doi:10.1523/JNEUROSCI.2226-22.2023)
Supplement: Table 1-1 — : Microarray analysis of differentially expressed microglia-related or -specific genes. Download Table 1-1, DOCX file. [file ns-JN-RM-2226-22-s01.docx]

**Extended Data Table 1-1**

| **Gene Symbol** | **Fold Change*** | **P-val** | **FDR P-val** | **Description** |
| --- | --- | --- | --- | --- |
| Gm10600 | 3.06 | 0.0003 | 0.0444 | predicted gene 10600 [Source:MGI Symbol;Acc:MGI:3710628] |
| Gm13304 | 2.28 | 0.0001 | 0.0299 | predicted gene 13304 |
| Gm10591 | 2.22 | 0.0001 | 0.0298 | predicted gene 10591 |
| Ccl21b; Gm13304 | 2.14 | 0.0003 | 0.0484 | chemokine (C-C motif) ligand 21B (leucine); predicted gene 13304 |
| Gm13304 | 2.14 | 0.0003 | 0.0484 | predicted gene 13304 |
| Epsti1 | -2.00 | 9.13E-05 | 0.022 | epithelial stromal interaction 1 (breast) |
| Entpd1 | -2.02 | 0.0001 | 0.027 | ectonucleoside triphosphate diphosphohydrolase 1 |
| Ehd4 | -2.1 | 3.23E-05 | 0.0109 | EH-domain containing 4 |
| Plcg2 | -2.11 | 8.72E-05 | 0.0215 | phospholipase C, gamma 2 |
| Lilr4b | -2.15 | 0.0002 | 0.0381 | leukocyte immunoglobulin-like receptor, subfamily B, member 4B |
| Il10ra | -2.15 | 0.0001 | 0.0273 | interleukin 10 receptor, alpha |
| Nfam1 | -2.16 | 6.31E-05 | 0.0166 | Nfat activating molecule with ITAM motif 1 |
| Cmklr1 | -2.17 | 0.0002 | 0.0444 | chemokine-like receptor 1 |
| Cyba | -2.18 | 2.49E-05 | 0.0091 | cytochrome b-245, alpha polypeptide |
| Folr2 | -2.2 | 6.57E-05 | 0.017 | folate receptor 2 (fetal) |
| Csf3r | -2.22 | 0.0001 | 0.029 | colony stimulating factor 3 receptor (granulocyte) |
| Slfn2 | -2.24 | 7.72E-06 | 0.0049 | schlafen 2 |
| Ccr5 | -2.24 | 5.67E-05 | 0.0155 | chemokine (C-C motif) receptor 5 |
| Il1a | -2.26 | 3.04E-05 | 0.0104 | interleukin 1 alpha |
| Tmem119 | -2.28 | 0.0002 | 0.0371 | transmembrane protein 119 |
| Mylip | -2.28 | 5.62E-05 | 0.0155 | myosin regulatory light chain interacting protein |
| Cxcl16 | -2.28 | 0.0002 | 0.0319 | chemokine (C-X-C motif) ligand 16 |
| Lcp2 | -2.32 | 0.0002 | 0.0444 | lymphocyte cytosolic protein 2 |
| B430306N03Rik | -2.33 | 4.64E-05 | 0.0132 | RIKEN cDNA B430306N03 gene |
| Ikzf1 | -2.34 | 0.0001 | 0.0283 | IKAROS family zinc finger 1 |
| Olfml3 | -2.36 | 0.0001 | 0.0253 | olfactomedin-like 3 |
| Rgs10 | -2.45 | 2.10E-05 | 0.0081 | regulator of G-protein signalling 10 |
| F13a1 | -2.53 | 3.96E-05 | 0.0122 | coagulation factor XIII, A1 subunit |
| Slamf9 | -2.61 | 0.0002 | 0.0319 | SLAM family member 9 |
| Tmem86a | -2.62 | 0.0002 | 0.0381 | transmembrane protein 86A |
| Nckap1l | -2.63 | 2.13E-05 | 0.0081 | NCK associated protein 1 like |
| Mnda | -2.64 | 2.28E-05 | 0.0086 | myeloid cell nuclear differentiation antigen |
| Slc2a5 | -2.65 | 0.0003 | 0.0444 | solute carrier family 2 (facilitated glucose transporter), member 5 |
| Rac2 | -2.68 | 1.49E-05 | 0.007 | RAS-related C3 botulinum substrate 2 |
| Ncf4 | -2.72 | 0.0001 | 0.0251 | neutrophil cytosolic factor 4 |
| Tyrobp | -2.77 | 6.36E-05 | 0.0166 | TYRO protein tyrosine kinase binding protein |
| Lilrb4a | -2.78 | 9.25E-05 | 0.0221 | leukocyte immunoglobulin-like receptor, subfamily B, member 4A |
| F11r | -2.78 | 7.24E-05 | 0.0185 | F11 receptor |
| Ifi30 | -2.79 | 4.51E-05 | 0.0131 | interferon gamma inducible protein 30 |
| Arhgdib | -2.79 | 3.86E-05 | 0.0121 | Rho, GDP dissociation inhibitor (GDI) beta |
| Ptafr | -2.81 | 0.0003 | 0.0484 | platelet-activating factor receptor |
| Unc93b1 | -2.83 | 1.79E-05 | 0.0076 | unc-93 homolog B1 (C. elegans) |
| Apoc2; Apoc4 | -2.87 | 0.0001 | 0.0298 | apolipoprotein C-II; apolipoprotein C-IV |
| Dock2 | -2.88 | 0.0002 | 0.0381 | dedicator of cyto-kinesis 2 |
| Cd14 | -2.9 | 9.00E-05 | 0.022 | CD14 antigen |
| Dock8 | -2.9 | 2.67E-05 | 0.0095 | dedicator of cytokinesis 8 |
| Havcr2 | -2.91 | 0.0001 | 0.0313 | hepatitis A virus cellular receptor 2 |
| Rbm47 | -2.98 | 2.69E-05 | 0.0095 | RNA binding motif protein 47 |
| Bcl2a1b; Bcl2a1a | -2.99 | 0.0002 | 0.0399 | B cell leukemia/lymphoma 2 related protein A1b; B cell leukemia/lymphoma 2 related protein A1a |
| Ncf1 | -3.04 | 1.58E-05 | 0.0072 | neutrophil cytosolic factor 1 |
| Cyth4 | -3.05 | 1.74E-06 | 0.002 | cytohesin 4 |
| Runx1 | -3.06 | 0.0001 | 0.0306 | runt related transcription factor 1 |
| Hexb | -3.1 | 1.88E-05 | 0.0078 | hexosaminidase B |
| C5ar1 | -3.13 | 1.64E-05 | 0.0073 | complement component 5a receptor 1 |
| Cd83 | -3.19 | 5.67E-05 | 0.0155 | CD83 antigen |
| Pik3cg | -3.19 | 4.54E-05 | 0.0131 | phosphoinositide-3-kinase, catalytic, gamma polypeptide |
| Vsir | -3.21 | 4.30E-05 | 0.0129 | V-set immunoregulatory receptor |
| P2ry13 | -3.22 | 0.0003 | 0.0444 | purinergic receptor P2Y, G-protein coupled 13 |
| Fcgr2b | -3.24 | 1.17E-05 | 0.0059 | Fc receptor, IgG, low affinity IIb |
| Pik3ap1 | -3.25 | 6.12E-05 | 0.0164 | phosphoinositide-3-kinase adaptor protein 1 |
| Igsf6 | -3.28 | 2.76E-06 | 0.0027 | immunoglobulin superfamily, member 6 |
| Abcc3 | -3.31 | 8.68E-05 | 0.0215 | ATP-binding cassette, sub-family C (CFTR/MRP), member 3 |
| AB124611 | -3.34 | 3.63E-06 | 0.0032 | cDNA sequence AB124611 |
| Adam8 | -3.36 | 0.0002 | 0.0381 | a disintegrin and metallopeptidase domain 8 |
| P2ry6 | -3.45 | 6.48E-06 | 0.0045 | pyrimidinergic receptor P2Y, G-protein coupled, 6 |
| Ms4a6d | -3.45 | 1.82E-06 | 0.002 | membrane-spanning 4-domains, subfamily A, member 6D |
| Ptgs1 | -3.49 | 4.92E-07 | 0.0014 | prostaglandin-endoperoxide synthase 1 |
| Tlr7 | -3.5 | 1.37E-05 | 0.0066 | toll-like receptor 7 |
| Fcgr3 | -3.53 | 3.77E-05 | 0.012 | Fc receptor, IgG, low affinity III |
| Ucp2 | -3.57 | 2.81E-05 | 0.0098 | uncoupling protein 2 (mitochondrial, proton carrier) |
| Slco2b1 | -3.64 | 2.39E-05 | 0.0088 | solute carrier organic anion transporter family, member 2b1 |
| Cd180 | -3.71 | 0.0003 | 0.0448 | CD180 antigen |
| Tbxas1 | -3.75 | 1.69E-06 | 0.002 | thromboxane A synthase 1, platelet |
| Ptpn6 | -3.76 | 3.78E-05 | 0.012 | protein tyrosine phosphatase, non-receptor type 6 |
| Lcp1 | -3.76 | 1.09E-06 | 0.002 | lymphocyte cytosolic protein 1 |
| Adgre1 | -3.85 | 1.95E-06 | 0.0021 | adhesion G protein-coupled receptor E1 |
| C1qa | -3.86 | 1.57E-05 | 0.0072 | complement component 1, q subcomponent, alpha polypeptide |
| AI607873 | -3.91 | 5.76E-05 | 0.0156 | expressed sequence AI607873 |
| Csf2rb2; Mir7676-1; Mir7676-2 | -3.95 | 4.32E-06 | 0.0033 | colony stimulating factor 2 receptor, beta 2, low-affinity (granulocyte-macrophage); microRNA 7676-1; microRNA 7676-2 |
| Ncf2 | -3.95 | 0.0002 | 0.0356 | neutrophil cytosolic factor 2 |
| Ccl12 | -4.05 | 9.18E-06 | 0.0051 | chemokine (C-C motif) ligand 12 |
| P2ry12 | -4.11 | 9.76E-05 | 0.0231 | purinergic receptor P2Y, G-protein coupled 12 |
| Tlr2 | -4.11 | 7.44E-07 | 0.0018 | toll-like receptor 2 |
| Cd72 | -4.18 | 3.91E-06 | 0.0032 | CD72 antigen |
| Samsn1 | -4.19 | 3.46E-05 | 0.0115 | SAM domain, SH3 domain and nuclear localization signals, 1 |
| Tgfb1 | -4.23 | 1.69E-06 | 0.002 | transforming growth factor, beta 1 |
| Inpp5d | -4.25 | 4.87E-06 | 0.0036 | inositol polyphosphate-5-phosphatase D |
| Gpr34 | -4.41 | 1.79E-06 | 0.002 | G protein-coupled receptor 34 |
| Rasgrp3 | -4.43 | 0.0001 | 0.0313 | RAS, guanyl releasing protein 3 |
| Cd68 | -4.52 | 9.46E-06 | 0.0051 | CD68 antigen |
| Itgam | -4.57 | 1.41E-07 | 0.0006 | integrin alpha M |
| Cd53 | -4.66 | 8.94E-07 | 0.002 | CD53 antigen |
| Ly86 | -4.67 | 1.37E-05 | 0.0066 | lymphocyte antigen 86 |
| Itgb2 | -4.67 | 0.0003 | 0.0484 | integrin beta 2 |
| Plek | -4.7 | 6.60E-06 | 0.0045 | pleckstrin |
| Aif1 | -4.8 | 1.13E-05 | 0.0058 | allograft inflammatory factor 1 |
| Lpcat2 | -4.81 | 2.38E-06 | 0.0024 | lysophosphatidylcholine acyltransferase 2 |
| Cybb | -5.00 | 4.09E-08 | 0.0005 | cytochrome b-245, beta polypeptide |
| Bin2 | -5.16 | 4.00E-06 | 0.0032 | bridging integrator 2 |
| Slc15a3 | -5.17 | 1.96E-05 | 0.0078 | solute carrier family 15, member 3 |
| Slc11a1 | -5.19 | 9.00E-06 | 0.0051 | solute carrier family 11 (proton-coupled divalent metal ion transporters), member 1 |
| Laptm5 | -5.25 | 3.72E-06 | 0.0032 | lysosomal-associated protein transmembrane 5 |
| Lyz2 | -5.33 | 1.12E-07 | 0.0006 | lysozyme 2 |
| Fcer1g | -5.44 | 1.93E-05 | 0.0078 | Fc receptor, IgE, high affinity I, gamma polypeptide |
| Tnf | -5.52 | 9.67E-08 | 0.0006 | tumor necrosis factor |
| Cd37 | -5.55 | 7.98E-05 | 0.0201 | CD37 antigen |
| Fcrls | -5.59 | 1.32E-06 | 0.002 | Fc receptor-like S, scavenger receptor |
| Mpeg1 | -5.62 | 6.65E-06 | 0.0045 | macrophage expressed gene 1 |
| Selplg | -5.7 | 4.01E-05 | 0.0122 | selectin, platelet (p-selectin) ligand |
| Cd52 | -5.74 | 1.33E-08 | 0.0003 | CD52 antigen |
| Cx3cr1 | -5.75 | 3.06E-06 | 0.0028 | chemokine (C-X3-C motif) receptor 1 |
| Tlr13 | -5.76 | 1.70E-05 | 0.0074 | toll-like receptor 13 |
| Pld4 | -5.91 | 1.63E-07 | 0.0006 | phospholipase D family, member 4 |
| Spi1 | -6.08 | 1.13E-06 | 0.002 | spleen focus forming virus (SFFV) proviral integration oncogene |
| Fermt3 | -6.08 | 4.39E-05 | 0.013 | fermitin family homolog 3 (Drosophila) |
| C1qb | -6.64 | 1.95E-05 | 0.0078 | complement component 1, q subcomponent, beta polypeptide |
| Trem2 | -6.93 | 1.46E-06 | 0.002 | triggering receptor expressed on myeloid cells 2 |
| Ctss | -7.08 | 3.05E-07 | 0.001 | cathepsin S |
| Gpr84 | -7.19 | 3.62E-05 | 0.0118 | G protein-coupled receptor 84 |
| C1qc | -7.53 | 8.17E-06 | 0.0049 | complement component 1, q subcomponent, C chain |
| Csf1r | -7.89 | 1.18E-06 | 0.002 | colony stimulating factor 1 receptor |
| Ccl3 | -8.34 | 7.01E-06 | 0.0046 | chemokine (C-C motif) ligand 3 |
| C3ar1 | -8.46 | 1.04E-05 | 0.0055 | complement component 3a receptor 1 |
| Spp1 | -9.64 | 8.48E-06 | 0.005 | secreted phosphoprotein 1 |
| Mmp12 | -19.27 | 8.20E-06 | 0.0049 | matrix metallopeptidase 12 |

*negative values indicate downregulation in PLX-treated tissue cultures
